# Designing for Dispersibility: How Crystallinity and Solubilizing Groups Affect Quantum Dot Dispersion in Diphenylhexatriene Matrices

**DOI:** 10.1021/acs.nanolett.5c05201

**Published:** 2026-01-19

**Authors:** Rachel C. Kilbride, Anastasia Leventis, Stephanie Montanaro, Ashish Sharma, James Xiao, Simon A. Dowland, Jurjen F. Winkel, Hugo Bronstein, Neil C. Greenham, Richard H. Friend, Akshay Rao, Oleksandr O. Mykhaylyk, Richard A. L. Jones, Anthony J. Ryan, Daniel T. W. Toolan

**Affiliations:** † Department of Physics, 2707 The University of Warwick, Coventry, CV4 7AL, U.K.; ‡ XMaS, The UK Materials Science Facility, European Synchrotron Radiation Facility, F-38043 Grenoble, France; § Yusuf Hamied Department of Chemistry, Cambridge University, Lensfield Road, Cambridge, CB2 1EW, U.K.; ∥ Cavendish Laboratory, University of Cambridge, J.J. Thomson Avenue, Cambridge, CB3 0HE, U.K.; ⊥ School of Mathematical and Physical Sciences, 7315 University of Sheffield, Brook Hill, Sheffield, S3 7HF, U.K.; # John Owens Building, 5292 The University of Manchester, Oxford Road, Manchester M13 9PL, U.K.; g Department of Materials, The University of Manchester, Engineering Building A, Booth Street East, Manchester M13 9PL, U.K.

**Keywords:** Quantum dots, Organic semiconductors, X-ray scattering, Singlet fission

## Abstract

Nanocomposite films combining organic semiconductors (OSCs) and colloidal quantum dots (QDs) are promising systems for next-generation optoelectronic technologies such as singlet-fission photon multiplication (SF-PM). Here, we show that tuning the solubilizing substituents on the high-triplet-energy SF-OSC (1*E*,3*E*,5*E*)-1,6-diphenylhexa-1,3,5-triene (DPH) enables precise control over film morphology and QD dispersibility. Grazing-incidence X-ray scattering reveals that PbS QDs ligated with oleic acid are poorly dispersed in all DPH derivatives, whereas hexanoic acid or DPH-carboxylic acid ligands significantly improve QD dispersibility. A clear design rule emerges: increasing solubilizing group volume relative to the DPH core enhances QD dispersibility, enabling well-dispersed QDs even in highly ordered DPH matrices. An exception arises in a derivative that forms an amorphous, nonequilibrium morphology that fully disperses QDs, but later crystallizes, resulting in QD aggregation. These findings show that OSC:QD nanocomposites require co-optimization of ligand–OSC chemistry and crystallization kinetics, providing a framework for designing efficient SF-PM and related technologies.

## Graphical abstract

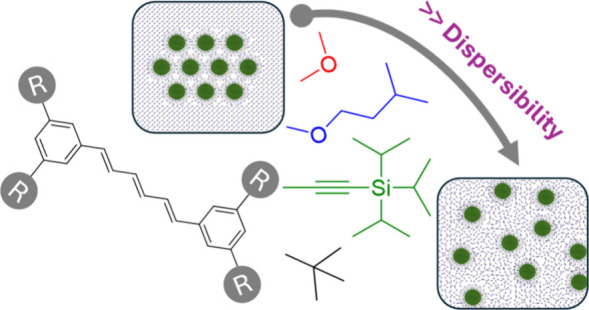

Nanocomposite films composed of colloidal quantum dots (QDs) and organic semiconductors (OSCs) have garnered significant attention for a wide range of optoelectronic applications, including solar cells, light-emitting diodes, and photon detectors.^1–5^ More recently, these systems have emerged as promising candidates for singlet-fission photon-multiplication (SF-PM),^6–8^ a novel approach to enhance the efficiency of silicon photovoltaics (Si-PV) beyond the theoretical limit imposed by thermodynamic constraints for a single-junction solar cell.^9^ This fundamental limit arises due to the excess energy that is lost as heat when high-energy photons with energies greater than the bandgap of the material are absorbed. The SF-PM concept relies on the conversion of these high-energy photons into multiple, lower energy photons via singlet fission to be effectively harnessed by an optically coupled Si-PV module. Such SF-PM systems have the potential to increase the theoretical efficiency limit of Si-PV upward from 33% to 44%.^6,7,10,11^ However, controlling the dispersion of QDs within the host OSC matrix is critical to optimizing SF-PM performance.

Previous work has demonstrated that the surface chemistry (i.e., ligands) of QDs is crucial in controlling the degree of QD aggregation or dispersion within the OSC matrix. For instance, our recent study on blade-coated blends of TIPS-tetracene (TIPS-Tc) and PbS QDs showed that when oleic acid (OA) is used as a QD ligand, the QDs form highly aggregated morphologies, leading to suboptimal performance. However, replacing OA with a chemically similar ligand, such as tetracene-carboxylic acid (TET-CA), significantly improves QD dispersion within the crystalline OSC matrix and leads to enhanced SF-PM performance.^8^ Further scattering and microscopy studies revealed complex QD morphologies, where QDs act as nucleation centers for OSC spherulites, some being distributed within the spherulite, while others are expelled to its periphery.^12–14^ Thus, the interplay between QD ordering and dispersibility with the crystallization of an OSC host is highly complex and is highly dependent upon interactions between the QD surface ligands and the OSC host, as well as the intrinsic crystallization behavior of the OSC.

While previous results on TIPS-Tc-based systems are highly promising, the relatively low triplet energies of such acenes (typically ∼0.81–1.25 eV^15,16^ ) are not ideally matched to couple with the bandgap of Si–PV (1.1 eV^17^). When accounting for energy losses in the triplet harvesting process, the ideal triplet energy of the OSC host in SF-PM systems is estimated to be ∼1.4–1.5 eV.^18^ There is therefore a need to explore alternative OSCs that meet the following requirements: (i) being capable of singlet fission with well-matched spectral characteristics to Si-PV and (ii) having good solution processability to ensure compatibility with large-scale deposition manufacturing techniques. Beyond SF-PM systems, it is crucial to establish universal design rules that will enable the generation of optimal QD morphologies within any host small-molecule organic semiconductor, which could pave the way for new conversion systems, LEDs, and photodetectors.

(1*E*,3*E*,5*E*)-1,6-Diphenylhexa-1,3,5-triene (DPH) and (1*E*,3*E*,5*E*)-1,6-bis­(1,3-dithian-2-yl)­hexa-1,3,5-triene (DTH) derivatives are highly promising OSCs capable of singlet fission with a triplet energy of ∼1.5 eV that if effectively harnessed would enable the SF-PM concept to be realized with Si-PV.^19–28^ The majority of SF studies on DPH derivatives have been restricted to molecules with poor solubility in common processing solvents.^19–25^ However, recently DPH/DTHs capable of SF with improved solubility have been achieved via the addition of solubilizing groups.^26–30^ In these studies, the molecular packing of the OSC plays a key role in determining the SF performance. While DPH- and DTH-based OSCs are both well spectrally matched with Si-PV and can be synthesized with a wide range of solubilizing groups that makes them compatible with large scale deposition manufacturing, studies have not yet focused on how QDs can be dispersed within these exciting SF materials in order to realize hybrid OSC:QD SF-PMF devices. A wide range of functional groups can be incorporated into DPH derivatives to enable solution processing, each likely influencing molecular packing and QD dispersibility in different ways. It is therefore essential to understand how they affect OSC:QD self-assembly, as such insights are crucial for optimizing the performance of SF-PM nanocomposite films.

In this study, we explore how different solubilizing groups influence the interactions between QD ligands and DPH-based OSCs, as well as the resulting crystallization behavior of the OSC. We synthesize a series of DPH derivatives with various solubilizing groups and blend them with PbS QDs functionalized with oleic acid (OA), hexanoic acid (C_6_), or a DPH-carboxylic acid analogue (Figure 1). Using grazing incidence X-ray scattering (GIXS), we characterize the self-assembly of these nanocomposite films, providing critical insights for optimizing SF-PM systems and other emerging optoelectronic applications. Achieving high-performance SF-PM nanocomposites demands a deeper understanding of how molecular structure governs the interplay between OSC crystallization and QD dispersion. By identifying key design rules that link solubilizing group chemistry to film morphology, this work aims to support the rational design of next-generation organic–inorganic hybrid materials for enhanced SF-PM and related optoelectronic applications.

**Figure 1:**
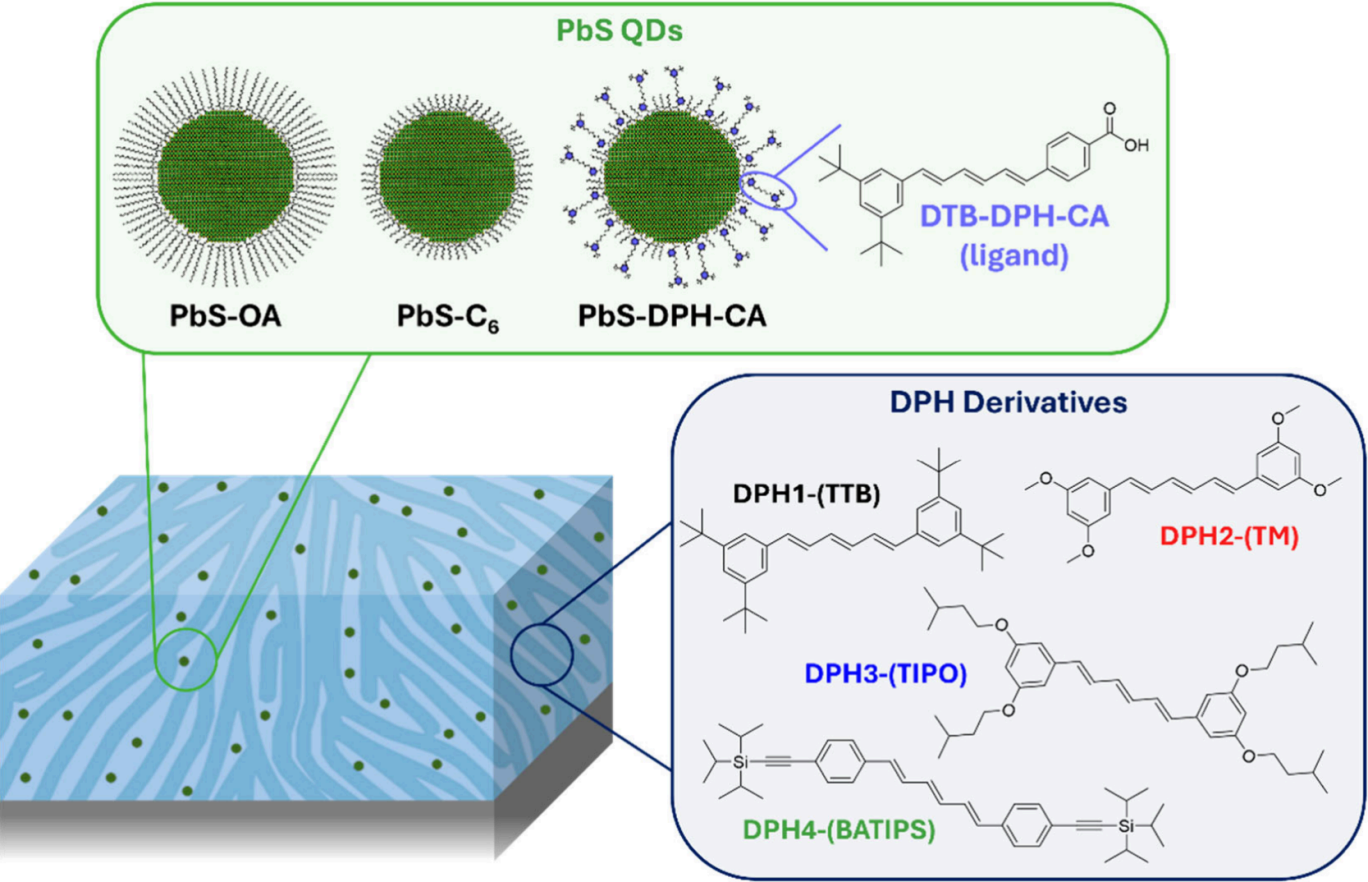
Schematic of QD:small molecule blend films, with PbS QDs [possessing either oleic acid (PbS-OA), hexanoic acid (PbS-C_6_), or a DPH-carboxylic acid derivative (PbS-DPH-CA) ligand] and various DPH small molecule host species [DPH1-(TTB), DPH2-(TM), DPH3-(TIPO), and DPH4-(BATIPS)].

Four different (1*E*,3*E*,5*E*)-1,6-diphenylhexa-1,3,5-triene (DPH) host derivatives were synthesized: (1*E*,3*E*,5*E*)-1,6-bis­(3,5-di-*tert*-butylphenyl)­hexa-1,3,5-triene [DPH1-(TTB)], (1*E*,3*E*,5*E*)-1,6-bis­(3,5-dimethoxy­phenyl)­hexa-1,3,5-triene [DHP2-(TM)], (1*E*,3*E*,5*E*)-1,6-bis­(3,5-bis­(isopentyloxy)­phenyl)­hexa-1,3,5-triene [DPH3-(TIPO)], and (1*E*,3*E*,5*E*)-1,6-bis­(4-((triisopropylsilyl)­ethynyl)­phenyl)­hexa-1,3,5-triene [DPH4-(BATIPS)] (Figure 1). These DPH derivatives were designed to systematically evaluate how different solubilizing groups influence QD exclusion from the DPH host, with the goal of identifying systems in which QDs remain highly dispersed within the matrix. Full synthesis details and NMR characterization are provided in Supporting Information Section S1 and Section S2, respectively.

The synthesis of the four DPH derivatives began with the preparation of tetraethyl but-2-ene-1,4-diyl­(*E*)-bis­(phosphonate) via a Michaelis–Arbuzov reaction between 1,4-dibromobut-2-ene and triethyl phosphite. This intermediate was subsequently used in a series of Horner–Wadsworth–Emmons reactions with commercially available aldehydes—3,5-di-*tert*-butylbenzaldehyde and 3,5-dimethoxy­benzaldehyde—to produce DPH1-(TTB) and DHP2-(TM), respectively. Similarly, reactions with custom-synthesized aldehydes—3,5-bis­(isopentyloxy)­benzaldehyde and 4-((triisopropylsilyl)­ethynyl)­benzaldehyde)— resulted in the formation of the corresponding derivatives, DPH3-(TIPO) and DPH4-(BATIPS). Single crystals of the DPH host materials (Supporting Information, Section S3) were successfully grown through solvent evaporation techniques with their structures determined by X-ray crystallographic analysis.

To complement the DPH host materials, a corresponding DPH carboxylic acid ligand, designed for direct interaction with the QD, was synthesized. The preparation of this ligand, DTB-DPH-CA, began with a Wittig reaction between (triphenylphosphoranylidene)­acetaldehyde and methyl-4-formylbenzoate. The resulting aldehyde was then reduced with sodium borohydride to form a primary alcohol, which was subsequently brominated by using phosphorus tribromide. A final Arbuzov reaction with triethyl phosphite produced methyl (*E*)-4-(3-(diethoxyphosphoryl)­prop-1-en-1-yl)­benzoate. This intermediate was then coupled with the custom-synthesized aldehyde (*E*)-3-(3,5-di-*tert*-butylphenyl)­acrylaldehyde to yield the DTB-DPH methyl ester, which was hydrolyzed under basic conditions with potassium hydroxide to afford the final ligand, DTB-DPH-CA.

PbS QDs were synthesized via a previously reported method^31^ with the as-synthesized native oleic acid (OA) ligands exchanged with either hexanoic acid (C_6_) or the matched DPH carboxylic acid derivative (DTB-DPH-CA) to obtain C_6_- and DPH-ligated PbS QDs (PbS-C_6_ and PbS-DPH-CA respectively) (full PbS synthesis and ligand exchange procedures are provided in Supplementary Section S4). The as-synthesized PbS-OA QDs were found via solution SAXS to have PbS cores measuring 16 Å in radius with a log-normal polydispersity of 0.14 (Supporting Information, Section S6). Based on previous works we predicted that both C_6_ and the matched DPH ligands would be expected to not only improve the dispersibility of the QDs within the functionalized DPH derivatives [DPH-(TTB-/TM-/TIPO-/BATIPS)] but also enable efficient triplet transfer from the DPH host to the QD.^8,13,32,33^ Composite DPH:QD films were produced via spin-coating, from toluene solutions [DPH-(TTB-/TM-/TIPO-/BATIPS)] (50 mg mL^–1^):PbS­(-OA/-C_6_/-DPH-CA) (10 mg mL^–1^) at 1,500 rpm on silicon substrates.

Grazing incidence X-ray scattering (GIXS) was performed on composite DPH:QD blend films to gain insight into the ordering of QDs within the film (at the small-angle region, GISAXS, in the *q* range 0.05–0.3 Å^–1^) and the small molecule packing/crystallinity (at the wide-angle region, GIWAXS, in the *q* range 0.3–1.7 Å^–1^) (Figure 2) where *q* = 4π sin θ/λ is the modulus of the scattering vector, θ is half of the scattering angle, and λ is the wavelength of the X-ray radiation. The collected two-dimensional scattering patterns clearly show that the combinations of different functionalized DPHs with PbS QDs (possessing either oleic acid, hexanoic acid, or a DPH-carboxylic acid derivative ligand) give rise to a wide variety of film morphologies. All DPH1-(TTB):QD blends exhibit a broad scattering feature in the high-*q* (GIWAXS) region, indicating a highly amorphous/disordered arrangement of these molecules. DPH4-(BATIPS):QD blends also exhibit weak intensity, broad scattering features, suggesting a highly disordered film. In contrast, DPH2-(TM):QD and DPH3-(TIPO):QD blends show distinct diffraction peaks associated with the formation of crystalline OSC structures, indicating greater small molecule ordering in these films. The GIWAXS data were compared with simulated powder X-ray diffraction (XRD) profiles of the single crystal structures (as determined in Supplementary Section S3). The DPH2-(TM):QD and DPH3-(TIPO):QD blend films were highly crystalline, and the diffraction peaks in the GIWAXS linecuts can be indexed to reflections in the single crystal X-ray profiles Figure S6. For DPH3-(TIPO):PbS-C_6_ and DPH3-(TIPO):PbS-DPH-CA blend films, the distinct preferential orientation can be replicated by simulating ordered packing with the 002 planes aligned parallel to the substrate (Figure S7). Interestingly, the GIWAXS data for DPH1-(TTB):QD blend films exhibit a distinctly different amorphous-type morphology, deviating from that of the equilibrium crystalline DPH1-(TTB) structure obtained via single crystal XRD.

**Figure 2:**
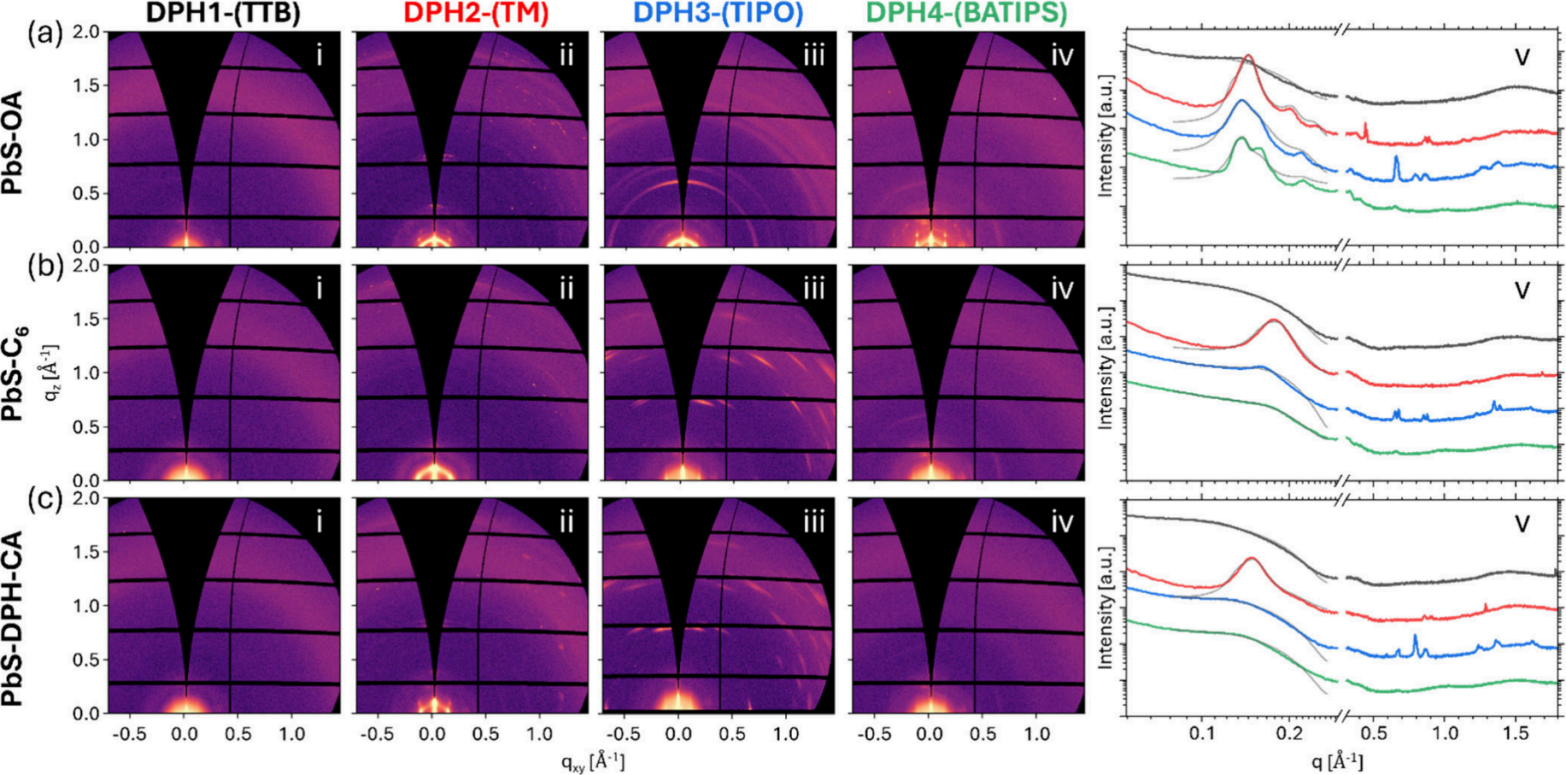
2D GISAXS patterns for blends comprising (a) PbS-OA, (b) PbS-C_6_, and (c) PbS-DPH-CA QDs with the following OSCs: (i) DPH1-(TTB), (ii) DPH2-(TM), (iii) DPH3-(TIPO), and (iv) DPH4-(BATIPS). (v) Corresponding 1D azimuthally integrated GISAXS and GIWAXS data are shown for DPH1-(TTB) (black), DPH2-(TM) (red), DPH3-(TIPO) (blue), and DPH4-(BATIPS) (green), along with fits to the GISAXS data (gray lines) using either an FCC paracrystal model (DPH1-(TTB), DPH3-(TIPO), DPH4-(BATIPS) QD blends) or a BCC paracrystal model (DPH2-(TM) QD blends). The 1D data were multiplied by arbitrary coefficients to shift them along the intensity axis for clarity.

The low *q* scattering region provides insight into the packing and ordering of QDs throughout the OSC matrix. Across the different DPH:QD systems, the scattering patterns reveal a wide range of QD dispersibilities within the various DPH matrices. At one extreme, films show well-ordered, closely packed QDs with a pronounced structure factor peak at *q* ∼ 0.15 Å^–1^ and scattering consistent with face-centered cubic (FCC) or body-centered cubic (BCC) superlattice features. At the other extreme, films exhibit more dispersed, randomly arranged QDs with scattering closely resembling the expected form factor of noninteracting spherical particles.

The dispersibility of the QDs in the DPH derivatives is characterized more quantitatively through fitting the low *q* region of the azimuthally integrated scattering data (Figure 2(a–c)­v). Here, the QD scattering data have been fitted using either an FCC or BCC paracrystal model (gray lines), with full fit parameters displayed in Table S3 (further information regarding BCC and FCC paracrystal models is provided in the Supporting Information Section S9). These models have been employed previously to describe QD ordering in blends comprising small molecule polyacenes and QDs.^8,14^ As shown in simulated 1D profiles (Supporting Information Section S9.3), the disorder parameter obtained from FCC and BCC colloidal models provides a direct measure for quantifying the QD dispersibility within the host DPH matrix, where low disorder parameters correspond to a highly ordered FCC or BCC arrangement of QDs (e.g., highly aggregated) and high disorder parameters correspond to weakly ordered QD arrangements (where a value of 1.0 is equivalent to randomly dispersed QDs that could be described by a scattering model of spheres with hard-sphere interactions).

For blends containing oleic acid ligated QDs (PbS-OA), the QDs were poorly dispersed across all DPH derivatives (low disorder parameter values). Here, DPH1-(TTB)/DPH3-(TIPO)/DPH4-(BATIPS):QD blends were fitted using an FCC paracrystal model, while the QD arrangement in DPH2-(TM):QD blends was more adequately represented using a BCC paracrystal model. The generation of BCC-packed QDs in the case of DPH2-(TM):QD blends likely arises from the incorporation of DPH2-(TM) within the QD-packed regions during the growth of the ordered BCC phase. This behavior is likely a consequence of DPH2-(TM) possessing solubilizing groups that are both small and relatively polar, which in turn influences the self-assembly of the system. How the self-assembly of DPH2-(TM):QD systems differs from that of other DPH derivatives will be the focus of future *in situ* GIWAXS experiments to fully understand this atypical behavior. For DPH2-(TM), changing the QD ligand from oleic acid to either hexanoic acid or DPH carboxylic acid resulted in only a small increase in the disorder parameter, indicating a modest improvement in QD dispersibility. In contrast, for DPH1-(TTB)/DPH3-(TIPO)/DPH4-(BATIPS):QD blends, ligand exchange resulted in a much larger increase in the disorder parameter, indicating substantial improvements in QD dispersibility in these systems (Table S3). Interestingly, we note that the dispersibility of PbS-C_6_ and PbS-DPH-CA is similar across all DPH derivatives, suggesting that it is not always necessary to chemically match the QD ligands to a specific functional group of the OSC to achieve good QD dispersibilities.

Although PbS-C_6_ and PbS-DPH-CA improve dispersibility in the DPH3-(TIPO):QD and DPH4-(BATIPS):QD blends, QD dispersibility is still comparable to that of PbS-OA in the DPH1-(TTB):QD system. Most notably, PbS-C_6_ and PbS-DPH-CA QDs in DPH1-(TTB) matrices yield exceptionally high disorder parameters approaching 1, consistent with fully dispersed QDs. We attribute the superior QD dispersibility in the DPH1-(TTB) matrix to the formation of an amorphous, highly disordered morphology. As the film dries and vitrifies, the QDs are likely trapped in place, preventing further self-assembly processes such as crystallization, which would otherwise expel QD impurities from growing crystallites, as reported in related systems.^12,13^

Upon remeasuring the DPH1-(TTB):QD films with GIWAXS after ∼2 weeks of storage under dark, ambient conditions, strong crystalline DPH1-(TTB) features are observed for DPH1-(TTB):PbS-OA and DPH1-(TTB):PbS-C_6_, with considerably weaker features observed for DPH1-(TTB):PbS-DPH-CA (Supporting Information, Section S11). This suggests that DPH1-(TTB):QD films are metastable with the initial amorphous morphology not fully quenched and prone to crystallization over time. The observed crystalline peaks do not match those expected for the bulk single crystal structure and indicate that DPH1-(TTB) is capable of forming multiple crystalline polymorphs (Figure S10). Fitting the 1D azimuthally integrated GISAXS profiles with an FCC paracrystal model reveals that films with increased DPH1-(TTB) crystallinity exhibit significantly reduced disorder parameters (Figure S9 and Table S4), consistent with QD aggregation over time as QDs are expelled from the growing OSC crystals.

To gain further insight into the trends in the fitted QD disorder parameter as a function of both the QD ligand and the host matrix, the ratio of the total volume of the solubilizing groups over the volume of the DPH core for all of the DPH derivatives is plotted against the QD disorder parameter (Figure 3, where molecule/functional group volumes were obtained using Molinspiration^34^). For DPH2-(TM), DPH3-(TIPO), and DPH4-(BATIPS) a correlation between increasing the volume of the solubilizing groups relative to that of the DPH core is obtained, whereby larger solubilizing groups lead to improved dispersibilities of PbS-C_6_ and PbS-DPH-CA QDs. However, DPH1-(TTB) is a significant outlier to this trend with the deviation attributed to the nonequilibrium amorphous type morphologies formed in the DPH1-(TTB):QD films. However, when DPH1-(TTB):PbS-C_6_ and DPH1-(TTB):PbS-DPH-CA films are aged, the crystallization of the OSC phase and subsequent increase in QD ordering results in a disorder parameter that is intermediate between DPH2-(TM):QD and DPH3-(TIPO)/DPH4-(BATIPS):QD blends (i.e., it falls into the trend that larger solubilizing groups correlate to improved QD dispersion) (Figure 3c).

**Figure 3:**
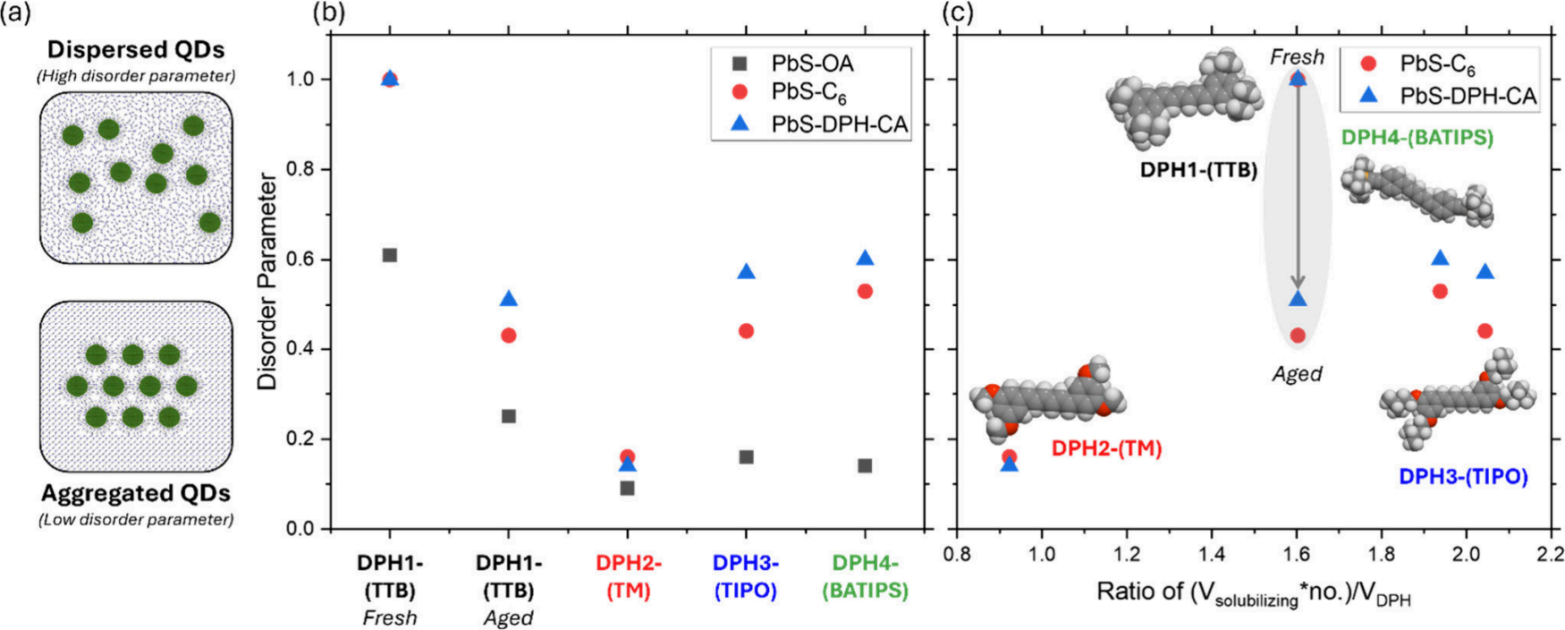
(a) An illustration of QD ordering in films with a high QD dispersibility (characterized by a high disorder parameter) and in films with a high degree of QD aggregation (characterized by a low disorder parameter). QD disorder parameter data derived from the fits to the GISAXS data for the QD:small molecule blend films for (b) the various DPH small molecule host species [DPH1-(TTB), DPH2-(TM), DPH3-(TIPO), and DPH4-(BATIPS)] and (c) as a function of the volume of the solubilizing groups/DPH core calculated for the various DPH small molecule host species. The gray shaded region shows the decrease in the disorder parameter upon film aging for DPH1-(TTB):PbS-C_6_ and DPH1-(TTB):PbS-DPH-CA blends due to DPH1-(TTB) crystallization and subsequent QD aggregation. The different QDs are labeled as follows: PbS-OA (gray squares), PbS-C_6_ (red circles), and PbS-DPH-CA (blue triangles).

We hypothesize that the trend of increasing QD dispersibility with increasing volume of solubilizing groups likely arises from lower solubility molecules, crystallizing from solution earlier in the film formation process, thus providing more time to exclude the QD “impurities” as the DPH molecules crystallize. DPH2-(TM), which contains the smallest solubilizing groups and yields highly crystalline films, likely initiates crystallization relatively early in the drying process. This early onset provides sufficient time for QD impurities to be excluded from the developing crystalline matrix. For DPH4-(BATIPS) and DPH3-(TIPO), the relative crystallinities differ markedly, with DPH3-(TIPO):QD films being significantly more crystalline than DPH4-(BATIPS):QD films. Despite the high crystallinity of DPH3-(TIPO), the disorder parameter for the matched PbS-DPH-CA QDs is similar to that in the far more disordered DPH4-(BATIPS) system. These observations highlight the complexity of achieving well-dispersed QDs in a small-molecule host. Even the strongly crystallizing DPH3-(TIPO) system shows comparatively good QD dispersibility, comparable to DPH4-(BATIPS). This suggests that, although DPH4-(BATIPS) processing has been tuned to limit crystallization during film formation, the modifications that achieve this also diminish favorable interactions with the QD ligands, yielding poorer dispersion than expected. By contrast, DPH1-(TTB) forms an amorphous host that crystallizes very slowly, allowing us to decouple crystallization kinetics from small-molecule:QD compatibility when assessing dispersion. DPH1-(TTB), as an amorphous host, is an attractive system to explore further, given its ability to disperse QDs effectively. Notably, once DPH1-(TTB) does crystallize, dispersibility falls to levels consistent with predictions based solely on solubilizing-group volume.

The observed behavior of the QD:DPH blend systems can be summarized and illustrated as the following (Figure 4): a highly crystalline DPH such as DPH2-(TM), irrespective of the QD ligand chemistry, demonstrates poor dispersibility of the QD within the DPH matrix, while for DPH1-(TTB), which formed an amorphous morphology, QDs could be homogeneously incorporated within the film, fully dispersed, with aggregation completely suppressed. These results confirm that small molecule crystallization is a significant driving force for the exclusion and consequent aggregation of QDs from a small molecule matrix. DPH4-(BATIPS) and DPH3-(TIPO) QD systems behave similarly to TIPS-tetracene and TIPS-anthracene type systems that we have studied previously,^8,12,13,32,33^ where matching the QD ligand chemistry to aspects of the host small molecule substantially improves QD dispersibility relative to the as-synthesized OA; however, some degree of aggregation persists within such samples, likely driven by the exclusion of QD impurities from the growing DPH crystalline matrix.

**Figure 4:**
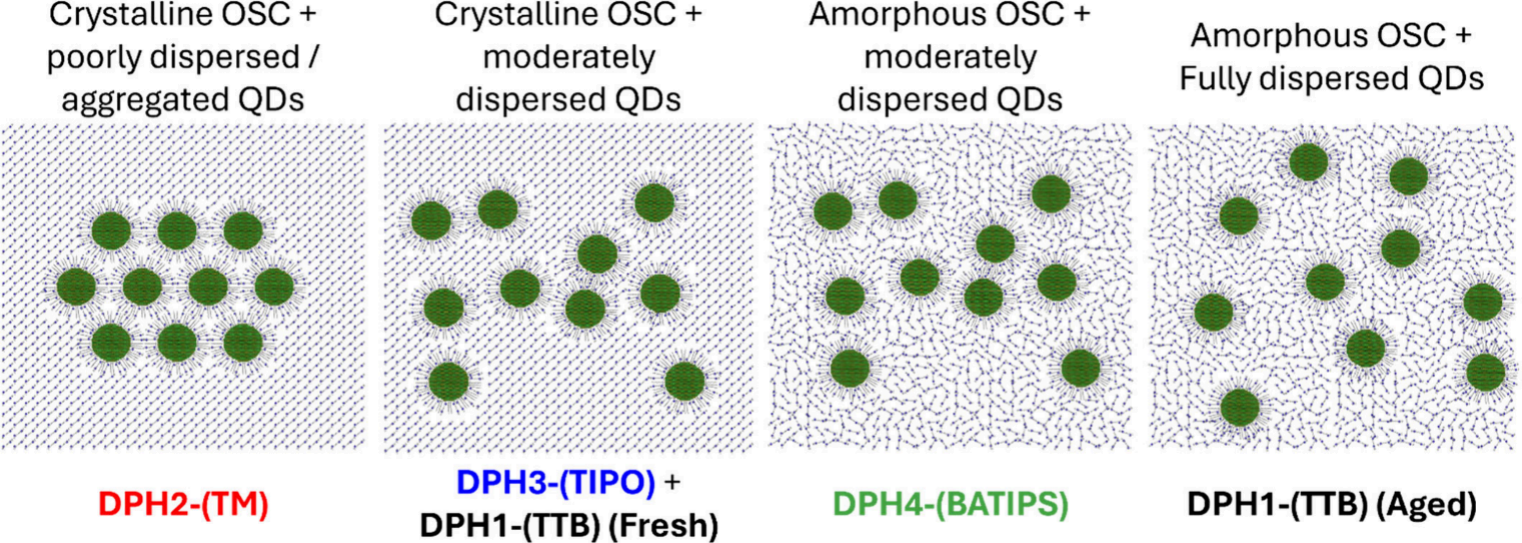
Illustration of the range of QD dispersibilities observed for the various functionalized DPH OSC materials explored in this work.

## 3 Conclusions

This work establishes how targeted molecular engineering of DPH solubilizing groups controls QD dispersibility in solution-processed DPH:QD films. Using PbS QDs with various ligands, three different effects have been identified:

- QDs ligated with oleic acid are consistently poorly dispersed; replacing oleic acid with hexanoic acid or a matched DPH-carboxylic-acid ligand significantly enhances QD dispersibility.
- Increasing the molecular volume of the solubilizing group relative to that of the DPH core systematically enhances QD dispersibility across the DPH series synthesized and studied here.
- Through the formation of nonequilibrium amorphous morphologies, it is possible to obtain dispersed QDs within a DPH host phase. However, it is found that such kinetically trapped systems may crystallize given sufficient time and that such crystallization reduces QD dispersibility and restores the trend of solubilizing group to DPH core volume ratio.

Taken together, this work demonstrates that QD dispersibility shows no simple correlation with the apparent crystallinity of the DPH OSC matrix. While crystallization dynamics drive QD exclusion, from the DPH host matrix, absolute crystallinity is not an adequate measure to predict QD dispersibility. The practical design rule obtained here is that both the solubilizing-group volume and ligand OSC interactions must be tuned to maximize ligand–matrix compatibility, while managing processing to moderate crystallization kinetics. These guidelines enable the generation of stable, well-dispersed OSC:QD nanocomposites for SF-PM and related optoelectronic technologies.

## Supplementary Material



## Data Availability

CCDC 2469741–2469744 contain the supplementary crystallographic data for this paper. These data can be obtained free of charge via www.ccdc.cam.ac.uk/data_request/cif, or by emailing data_request@ccdc.cam.ac.uk, or by contacting The Cambridge Crystallographic Data Centre, 12 Union Road, Cambridge CB2 1EZ, UK; fax: + 44 1223 336033.

## Notes

Experimental methods, synthesis, crystal structures, solution SAXS, 2D GIWAXS simulations, 1D GISAXS fitting models and parameters, aged film data (PDF)
