# Supplementary material for: Designing for Dispersibility: How Crystallinity and Solubilizing Groups Affect Quantum Dot Dispersion in Diphenylhexatriene Matrices
Source: Nano Lett. 2026 Jan 19;26(4):1274–80. doi: 10.1021/acs.nanolett.5c05201 (PMC13003486; doi:10.1021/acs.nanolett.5c05201)
Supplement: Supplementary file 1 [file nl5c05201_si_001.pdf]

Supporting Information

Designing for Dispersibility: How Crystallinity and Solubilising Groups Affect Quantum Dot Dispersion in Diphenylhexatriene Matrices

Rachel C. Kilbride[a,b], Anastasia Leventis[c], Stephanie Montanaro[c], Ashish Sharma[d], James Xiao[d], Simon A. Dowland[d], Jurjen F. Winkel[d], Hugo Bronstein[c], Neil C. Greenham[d], Richard H. Friend[d], Akshay Rao[d], Oleksandr O. Mykhaylyk[e], Richard A. L. Jones[f], Anthony J. Ryan[e] and Daniel T. W. Toolan[g]*

a) Department of Physics, The University of Warwick, Coventry, CV4 7AL, U.K.
b) XMaS, The UK Materials Science Facility, European Synchrotron Radiation Facility, F-38043 Grenoble, France.
c) Yusuf Hamied Department of Chemistry, Lensfield Road, Cambridge, CB2 1EW, U.K.
d) Cavendish Laboratory, University of Cambridge, J.J. Thomson Avenue, Cambridge, CB3 0HE, U.K.
e) School of Mathematical and Physical Sciences, University of Sheffield, Brook Hill, Sheffield, S3 7HF, U.K.
f) John Owens Building, The University of Manchester, Oxford Road, Manchester M13 9PL, U.K.
g) Department of Materials, University of Manchester, Engineering Building A, Booth Street East, Manchester M13 9PL, U.K.

Corresponding author: daniel.toolan@manchester.ac.uk

Keywords: Quantum dots, Organic semiconductors, X-ray scattering, singlet fission

Contents

S1 – DPH Derivative Synthesis ... 3

S1.1 - General Experimental Information ... 3

S1.2 - Synthesis of DPH Host Materials ... 4

S1.3 - Synthesis of DTB-DPH-CA ... 11

S2 - 1H NMR and 13C NMR Spectra ... 18

S3: Single Crystal Structures of the DPH Derivatives ... 32

S3.1 - Single-crystal X-ray diffraction ... 33

S4- Synthesis and Exchange of Lead-Sulphide QDs ... 35

S4.1. Synthesis of PbS-OA QDs ... 35

S4.2. Exchange of PbS-OA to PbS-DPH-CA and PbS-C_6 ... 35

S5: Experimental Methods ... 36

S5.1. Film preparation ... 36

S5.2. X-ray Scattering ... 36

S6: Solution SAXS ... 37

S7: Single Crystal and Thin Film Crystal Structure Comparison ... 38

S8: 2D GIWAXS Simulations ... 39

S9: Colloidal Paracrystal Models ... 40

S9.1 - FCC Paracrystal Model ... 40

S9.2 - BCC Paracrystal Model ... 40

S9.3 – Simulated FCC and BCC 1D Profiles ... 41

S10: 1D GISAXS Fitting ... 42

S11: Aged DPH1-(TTB):QD Films ... 43

S12: References ... 45

S1 – DPH Derivative Synthesis

S1.1 - General Experimental Information

All reactions were carried out in oven-dried flasks under an inert argon atmosphere and covered with foil unless otherwise indicated, and all reactions were stirred magnetically. Anhydrous (anh.) solvents were used under an inert argon atmosphere. All other chemicals were used as supplied. Column chromatography was carried out using Supelco® Silica gel 60 (40-63 μm) or using a Biotage® Isolera™ One with Biotage® Sfär Silica D - Duo 60 μm cartridges (10 g, 20 g, 50 g or 100 g). Analytical thin layer chromatography was carried out using Silica gel coated aluminium TLC sheets. Components were visualised using ultra-violet light. ^{1}H NMR spectra were recorded at 400 MHz on a Bruker Advance III 400 spectrometer, at 500 MHz on a Bruker Advance III 500 spectrometer or at 700 MHz on a Bruker Advance III 700 Cryo spectrometer in the stated solvent using residual protic solvent $CHCl_3$ (δ = 7.26 ppm, s) and DMSO (δ = 2.50 ppm, s) as the internal standard. ^{1}H NMR chemical shifts are reported to the nearest 0.01 ppm and quoted using the following abbreviations: s, singlet; d, doublet; t, triplet; q, quartet; qn, quintet; sxt, sextet; m, multiplet; br, broad; Ar, aromatic or a combination of these. The coupling constants (*J*) are measured in Hertz. ^{13}C NMR spectra were recorded at 150 MHz on a spectrometer in the stated solvent using the central reference of $CHCl_3$ (δ= 77.16 ppm, t) and $(CD_3)_2SO$ (δ = 39.52 ppm, s) as the internal standard. ^{13}C NMR chemical shifts are reported to the nearest 0.1 ppm. Mass spectra were obtained using an Agilent LC-TOF/TQD/GCMS at the Yusuf Hamied Department of Chemistry, University of Cambridge.

S1.2 - Synthesis of DPH Host Materials

Tetraethyl but-2-ene-1,4-diyl(E)-bis(phosphonate)

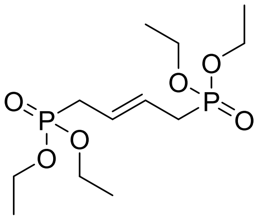

Procedure from[1,2]

1,4-dibromo-2-butene (10.0 g, 46.7 mmol, 1 equiv.) and triethyl phosphite (19 mL, 111 mmol, 2.4 equiv.) were added to an oven-dried flask set up for reflux under argon atmosphere. The mostly dissolved mixture was heated to 160 °C and allowed to stir for 20 h. The resulting mixture was cooled to RT, dry loaded onto silica and purified by flash column chromatography (Chloroform: Acetone, 5:1), to give the title product as a clear oil, (8.81 g, 57%)

R_f: 0.15 (Chloroform: Acetone, 5:1); **^{1}H NMR** (600 MHz CDCl$_3$) δ (ppm): 5.56 (m, 2H, C*H*=C*H*), 4.09 – 4.02 (m, 8H, C*H*$_2$CH$_3$), 2.56 (ddd, *J* = 17.6, 4.2, 1.7 Hz, 4H, C*H*$_2$CH), 1.27 (t, *J* = 7.1 Hz, 12H, C*H*$_3$). **^{13}C NMR** (151 MHz, CDCl$_3$): 124.4, 124.4, 124.4, 62.0, 62.0, 62.0, 31.1, 31.1, 30.2, 30.2, 16.5, 16.5, 16.5. **HRMS**: (FTMS + p ESI): Calculated for $C_{12}H_{27}O_6P_2$: 329.1277 [M+H]$^+$. Found *m/z* 329.1267 [M+H]$^+$.

Spectroscopic data supported by the literature.[3]

(1E,3E,5E)-1,6-Bis(3,5-di-tert-butylphenyl)hexa-1,3,5-triene

DPH1-(TTB)

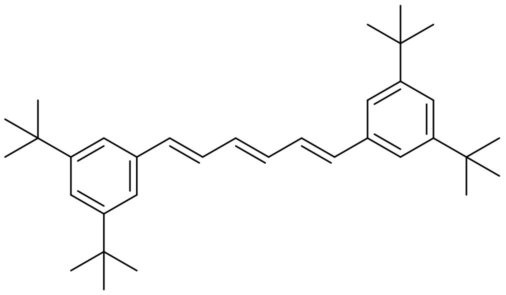

Tetraethyl but-2-ene-1,4-diyl(E)-bis(phosphonate) (0.53 g, 1.61 mmol, 1.0 equiv.) and anh. THF (7.5 mL) were added to an oven-dried flask under argon. Sodium hydride (0.045 g, 1.88 mmol, 1.2 equiv.) was then added, followed by more anh. THF (1.9 mL), and the resulting mixture was stirred at RT for 30 mins (cloudy cream mixture). A pre-dissolved solution of 3,5-di-tert-butylbenzaldehdye (0.84 g, 3.85 mmol, 2.4 equiv.) in anh. THF (7 mL), was then added dropwise over 5 mins and then left to stir for 30 mins at RT. A colour change was observed from cloudy cream to a translucent orange solution. A second dose of sodium hydride (0.041 g, 1.71 mmol, 1.1 equiv.) was added, followed by anh. THF (1.9 mL), which was then left to stir at RT for 24 h. The resulting orange solution was hydrolysed with water and then extracted with diethyl ether (1M HCl solution can be added to help separate layers). The organic phases were separated and dried over $MgSO_4$, filtered through cotton wool, and concentrated *in vacuo*. The crude product was dry loaded onto silica and purified *via* column chromatography (Hexane: DCM (9:1). Fractions containing the product were combined and concentrated *in vacuo*, before being recrystallised from DCM:MeOH to afford the title product as a shiny yellow crystals (0.20 g, 27%).

R_f: 0.74 (Hexane: DCM, 2:1); **^{1}H NMR** (600 MHz $CDCl_3$) δ (ppm): 7.30 (t, *J* = 1.8 Hz, 2H, ArC*H*), 7.25 (d, *J* = 5.9 Hz, 4H, ArC*H*), 6.88 (ddd, *J* = 15.5, 7.1, 3.0 Hz, 2H, C*H*=C*H*), 6.61 (d, *J* = 15.5 Hz, 2H, C*H*=C*H*), 6.52 (dd, *J* = 7.1, 3.0 Hz, 2H, C*H*=C*H*), 1.34 (s, 36H, C*H*$_3$). **^{13}C NMR** (151 MHz, $CDCl_3$): 151.03, 136.69, 133.56, 133.40, 128.71, 122.09, 120.76, 34.89, 31.50. **HRMS**: (FTMS + p ESI): Calculated for $C_{34}H_{49}$: 457.3829 $[M+H]^+$. Found *m*/*z* 457.3824 $[M+H]^+$. **Single Crystal XRD**: Obtained.

(1E,3E,5E)-1,6-Bis(3,5-dimethoxyphenyl)hexa-1,3,5-triene

DPH2-(TM)

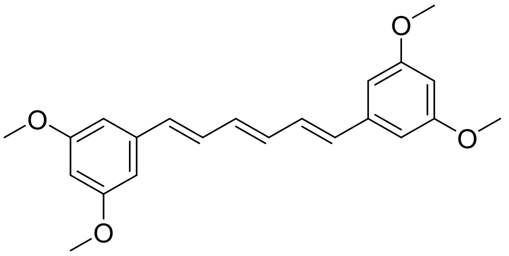

Tetraethyl but-2-ene-1,4-diyl(E)-bis(phosphonate) (1.0 g, 3.05 mmol, 1.0 equiv.) and anh. THF (14 mL) were added to an oven-dried flask under argon. Sodium hydride (0.079 g, 3.29 mmol, 1.1 equiv.) was then added, followed by more anh. THF (3.6 mL), and the resulting mixture was stirred at RT for 30 mins (cloudy cream mixture). A pre-dissolved solution of 3,5-dimethoxybenzaldehyde (1.47 g, 8.84 mmol, 2.9 equiv.) in anh. THF (14 mL), was then added dropwise over 5 mins and then left to stir for 30 mins at RT. A colour change was observed from cloudy cream to a partially cloudy, light orange solution. A second dose of sodium hydride (0.079 g, 3.29 mmol, 1.1 equiv.) was added, followed by anh. THF (3.6 mL), which was then left to stir at RT for 24 h. The resulting orange solution was hydrolysed with water and then extracted with diethyl ether (1M HCl solution can be added to help separate layers). The organic phases were separated and dried over $MgSO_4$, filtered through cotton wool, and concentrated *in vacuo*. The crude product was dry loaded onto silica and purified *via* column chromatography (Hexane: DCM (2:1). Fractions containing the product were combined and concentrated *in vacuo*, before being recrystallised from DCM:MeOH to afford the title product as a shiny yellow crystals (0.28 g, 26%).

R$_f$: 0.7 (DCM); **^{1}H NMR** (600 MHz $CDCl_3$) δ (ppm): 6.86 (ddd, *J* = 15.5, 7.0, 3.1 Hz, 2H, C*H*=CH), 6.59 (d, *J* = 2.2 Hz, 4H, Ph*H*), 6.54 (d, *J* = 15.5 Hz, 2H, C*H*=CH), 6.53 – 6.50 (m, 2H, C*H*=CH), 6.38 (t, *J* = 2.2 Hz, 2H, Ph*H*), 3.82 (s, 12H, OCH_3). **^{13}C NMR** (151 MHz $CDCl_3$) δ (ppm): 160.9, 139.3, 133.7, 132.8, 129.6, 104.4, 100.1, 55.3. **HRMS**: (TOF MS ASAP+): Calculated for $C_{22}H_{25}O_4$: 353.1675 $[M+H]^+$. Found *m/z* 353.1679 $[M+H]^+$. **Single Crystal XRD:** Obtained.

3,5-Bis(isopentyloxy)benzaldehyde

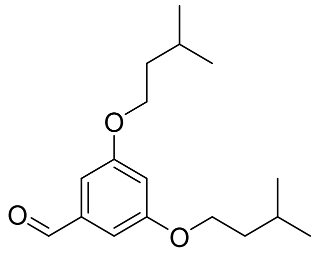

3,5-dihydroxybenzaldehyde (2.52 g, 18.2 mmol, 1.0 equiv.), potassium carbonate (8.2 g, 60.1 mmol, 3.3 equiv.), 1-bromo-3-methyl butane (9.8 mL, 81.8 mmol, 4.5 equiv.) and anh. DMF (21 mL) were added to an oven-dried flask under an argon atmosphere. The mixture was stirred at 55 ^{o}C for 2.5 h. The dark-red/orange/brown solution was then cooled to RT before water was added. The aqueous layer (brown) was separated and extracted with ethyl acetate (x3). The organic layers (orange/bright red) were combined and dried over $MgSO_4$, filtered through cotton, and concentrated *in vacuo*. The resulting wine-red oil was purified by flash column chromatography (1:1 Hexane: DCM initially, then 1:2 Hex: DCM) to afford the title product as a clear oil, (slight orange tinge) (4.4 g, 87%).

R$_f$: 0.5 (Hexane: DCM, 1:1); **^{1}H NMR** (400 MHz CDCl$_3$) δ (ppm): 9.88 (s, 1H, *H*C=O), 6.98 (d, *J* = 2.3 Hz, 2H, Ph-C*H*), 6.69 (t, *J* = 2.3 Hz, 1H, Ph-C*H*), 4.01 (t, *J* = 6.7 Hz, 4H, O-C*H*$_2$), 1.78-1.88 (m, 2H, CH$_2$C*H*), 1.68 (q, *J* = 6.7 Hz, 4H, CH$_2$C*H*$_2$CH), 0.96 (d, J = 6.6 Hz, 12H, C*H*$_3$). **^{13}C NMR** (100 MHz, CDCl$_3$): 192.1, 160.8, 138.3, 108.0, 107.6, 66.8, 37.9, 25.1, 22.6. **HRMS**: (TOF MS ASAP+): Calculated for $C_{17}H_{27}O_3$: 279.1960 $[M+H]^+$. Found *m/z* 279.1962 $[M+H]^+$.

(1E,3E,5E)-1,6-Bis(3,5-bis(isopentyloxy)phenyl)hexa-1,3,5-triene

DPH3-(TIPO)

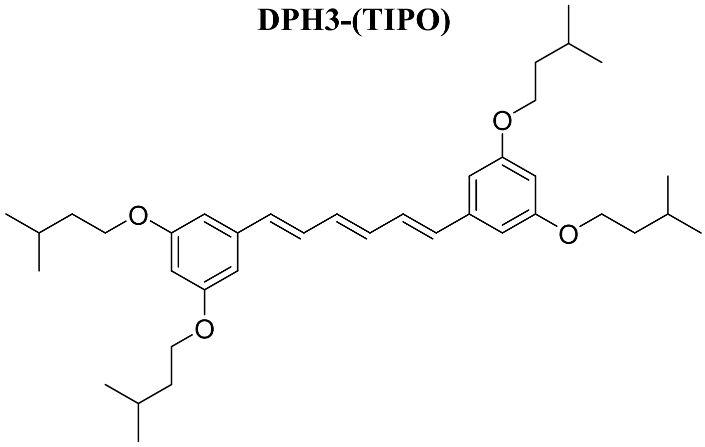

Tetraethyl but-2-ene-1,4-diyl(E)-bis(phosphonate) (0.40 g, 1.20 mmol, 1.0 equiv.) and anh. THF (5.7 mL) were added to an oven-dried flask under an argon atmosphere. Sodium hydride (0.034 g, 1.42 mmol, 1.2 equiv.) was then added, followed by more anh. THF (1.5 mL), and the resulting mixture was stirred at RT for 30 mins (cloudy cream mixture). A pre-dissolved solution of 3,5-bis(isopentyloxy)benzaldehyde (1.03 g, 3.70 mmol, 3.1 equiv.) in anh. THF (6.0 mL), was then added dropwise over 5 mins and then left to stir for 30 mins at RT. A colour change was observed from cloudy cream to a pale orange solution. A second dose of sodium hydride (0.034 g, 1.42 mmol, 1.2 equiv.) was added, followed by anh. THF (1.5 mL), which was then left to stir at RT for 24 h. The resulting orange solution was hydrolysed with water and then extracted with diethyl ether. The organic phases were separated and dried over MgSO4, filtered through cotton wool, and concentrated in vacuo. The crude product (viscous yellow oil) was dry loaded onto silica and purified via column chromatography (Hexane initially, then Hexane: DCM (2:1)). Two separate fractions containing the product were separately concentrated *in vacuo*. To F1 (a yellow oil), methanol was added, followed by a small amount of DCM. The solution was sonicated, and a pale yellow solid precipitated, which was filtered under vacuum to afford the pure, title product (0.17 g). The same process was repeated for F2, however the NMR was impure. Therefore, the residue was recrystallised from DCM:MeOH at RT to afford the pure, title product as pale yellow flakes (0.029 g), (Total yield of F1&F2: 0.20 g, 29%).

^{1}H NMR (700 MHz $CDCl_3$) δ (ppm): 6.84 (dd, J = 15.8, 7.9 Hz, 2H, C*H*=CH), 6.56 (s, 4H, Ph-C*H*), 6.51 (d, J = 15.8 Hz, 2H, C*H*=CH), 6.49 (m, 2H, C*H*=C*H*), 6.36 (m, 2H, Ph-C*H*), 3.99

(t, J = 6.6 Hz, 8H, O-CH_2), 1.84 (dp, J = 13.3, 6.7 Hz, 4H, CH_3CH), 1.68 (q, J = 6.7 Hz, 8H, CHC$H_2$$CH_2$), 0.97 (d, J = 6.7 Hz, 24H, CH_3). **^{13}C NMR** (176 MHz, $CDCl_3$): 160.5, 139.2, 133.6, 132.9, 129.5, 105.0, 101.0, 66.4, 38.0, 25.1, 22.6. **HRMS**: (TOF MS ASAP+): Calculated for $C_{38}H_{57}O_4$: 577.4257 $[M+H]^+$. Found m/z 577.4260 $[M+H]^+$. **Single Crystal XRD:** Obtained.

4-((Triisopropylsilyl)ethynyl)benzaldehyde

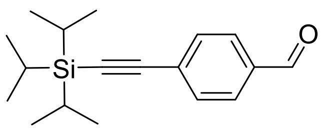

Procedure from[4]

4-bromobenzaldehyde (2.0 g, 10.8 mmol, 1 equiv.), (triisopropylsilyl)acetylene (3 mL, 13.4 mmol, 1.24 equiv.), copper (I) iodide (0.0142 g, 0.0746 mmol, 0.0067 equiv.), bis(triphenylphosphine)palladium(II) dichloride, $PdCl_2(PPh_3)_2$ (0.0513 g, 0.0731 mmol, 0.0067 equiv.) and triphenylphosphine (0.0471 g, 0.180 mmol, 0.016 equiv.) were degassed for 1 h and added to an oven-dried flask set up for reflux under argon atmosphere. Stock solutions of triethylamine (30 mL) and anh. THF (35 mL) were also degassed for 1 h. The degassed anh. THF (20 mL) and triethylamine (20 mL, 13.4 mmol, 1.24 equiv.) were added to the reagents, creating an initially yellow solution which turned clear after 20 mins at RT. The solution was then heated to 65 ^{o}C for 12 h. The resulting brown mixture was cooled to RT, and filtered under vacuum to remove solids, and the filtrate was diluted with DCM. The solution was washed with aq. ammonium chloride solution and the organic layers were extracted with DCM, dried over MgSO4, filtered through cotton and concentrated *in vacuo*. The crude, brown residue was purified by column chromatography (Hexane: DCM, 2:1), to give the title product as a clear oil, (1.9 g, 61%).

R$_f$: 0.3 (Hexane: DCM, 2:1); **^{1}H NMR** (600 MHz $CDCl_3$) δ (ppm): 10.00 (s, 1H, HC=O), 7.85 – 7.79 (m, 2H, PhH), 7.64 – 7.57 (m, 2H, PhH), 1.17 – 1.10 (m, 21H, CH_3CH). **^{13}C NMR** (151

MHz $CDCl_3$) δ (ppm): 191.4, 135.5, 132.5, 129.7, 129.4, 105.9, 95.8, 18.6, 11.2. **HRMS**: (Vion IMS QTOF, MsE): Calculated for $C_{18}H_{26}OSi$: 286.17529 $[M]^+$. Found *m/z* 287.18257 $[M+H]^+$.

Spectroscopic data supported by the literature.[4]

(1E,3E,5E)-1,6-Bis(4-((triisopropylsilyl)ethynyl)phenyl)hexa-1,3,5-triene

DPH4-(BATIPS)

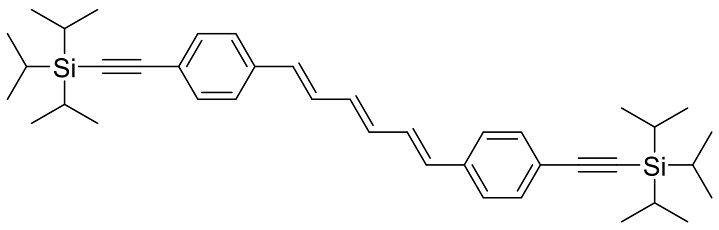

Tetraethyl but-2-ene-1,4-diyl(E)-bis(phosphonate) (0.79 g, 2.41 mmol, 1.0 equiv.) and anh. THF (11 mL) to an oven-dried flask under argon. Sodium hydride (60% wt. dispersion in mineral oil, 0.064 g, 2.67 mmol, 1.1 equiv.) was then added, followed by some more anh. THF (2.9 mL), and the resulting mixture was stirred at RT for 30 mins. A pre-dissolved solution of 4-((triisopropylsilyl)ethynyl)benzaldehyde (1.76 g, 6.14 mmol, 2.5 equiv.) in anh. THF (11 mL), was then added dropwise over 5 mins and then left to stir for 30 mins at RT. A colour change was observed from cloudy cream to a yellow/brown solution. A second dose of sodium hydride (60% wt. dispersion in mineral oil, 0.062 g, 2.58 mmol, 1.1 equiv.) was added, followed by anh. THF (2.9 mL), which was then left to stir at RT for 24 h. The resulting solution was hydrolysed with water and then extracted with diethyl ether. The organic phases were separated and dried over MgSO4, filtered through cotton wool, and concentrated *in vacuo*. The crude product (viscous orange oil) was dry loaded onto silica and purified via column chromatography (Hexane initially, then Hexane: DCM (2:1)). The fractions containing the product were concentrated *in vacuo*, and the residue was recrystallized from a mixture of DCM: Methanol (1:1) at RT over 12 h. Bright, fluorescent yellow crystals formed, which were collected after being filtered under vacuum and washed with methanol, (0.34 g, 24%).

^{1}H NMR (500 MHz $CDCl_3$) δ (ppm): 7.43 (dd, *J* = 8.2, 1.9 Hz, 4H, Ph*H*), 7.39 – 7.29 (m, 4H, Ph*H*), 6.89 (ddd, *J* = 15.5, 7.2, 3.1 Hz, 2H, C*H*=CH), 6.62 – 6.49 (m, 4H, C*H*=CH), 1.14 (m,

42H, C*H*$_3$C*H*). **^{13}C NMR** (126 MHz, $CDCl_3$): 137.3, 134.0, 132.4, 132.3, 129.9, 126.1, 122.5, 107.2, 91.7, 18.7, 11.4. **HRMS**: (FTMS + c ESI): Calculated for $C_{40}H_{56}Si_2$: 592.3915 $[M]^+$. Found *m/z* 592.3887 $[M]^+$. **Single Crystal XRD:** Obtained.

S1.3 - Synthesis of DTB-DPH-CA

Methyl (*E*)-4-(3-oxoprop-1-en-1-yl)benzoate

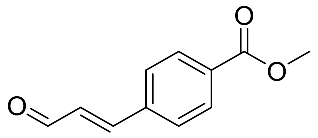

(Triphenylphosphoranylidene)acetaldehyde (60.0 g, 197 mmol, 1 equiv.), methyl-4-formyl benzoate (48.6 g, 296 mmol, 1.5 equiv.) and anh. toluene (1.2 L) were added to an oven-dried flask under argon. The mixture was stirred at 85 ^oC for 24 h. The crude mixture was purified via column chromatography, (eluent Hexane: DCM (4:1), then gradually to (2:1)). Fractions containing the product were combined and concentrated *in vacuo*. The resulting solid was sonicated in hexane and filtered under vacuum to afford the title product as a white flocculent powder (23.8 g, 64%).

^{1}H NMR (600 MHz $CDCl_3$) δ (ppm): 9.75 (d, J = 7.6 Hz, 1H, COC*H*), 8.12 – 8.07 (m, 2H, Ph*H*), 7.66 – 7.61 (m, 2H, Ph*H*), 7.50 (d, J = 16.0 Hz, 1H, C*H*=CH), 6.78 (dd, J = 16.0, 7.6 Hz, 1H, COCH-C*H*=CH), 3.94 (s, 3H, OC*H*$_3$). **^{13}C NMR** (151 MHz, $CDCl_3$): 193.6, 166.6, 151.2, 138.4, 132.6, 130.8, 130.6, 128.7, 52.8. **HRMS**: (TOF MS ASAP+): Calculated for $C_{11}H_{11}O_3$ 191.0708 $[M+H]^+$. Found *m/z* 191.0709 $[M+H]^+$.

Methyl (*E*)-4-(2-hydroxyvinyl)benzoate

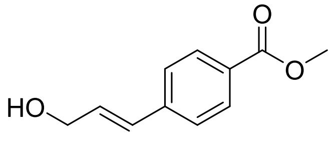

Methyl (*E*)-4-(3-oxoprop-1-en-1-yl)benzoate (1.00 g, 5.26 mmol, 1 equiv.), anh. THF (2 mL) and anh. methanol (6 mL) were added to an oven-dried flask under argon. The mixture was cooled to 0 °C before sodium borohydride (0.41 g, 10.8 mmol, 2.1 equiv), was added portion-wise. The resulting mixture was allowed to stir at RT for 2h. A colour change was observed from cloudy white to clear and colourless. After complete consumption of the starting material, the mixture was quenched with saturated ammonium chloride, and the solvent volume was reduced under vacuum. The residue left behind was extracted with ethyl acetate and the organic layer was separated, dried over $MgSO_4$, filtered and concentrated *in vacuo* to yield the allylic alcohol. This was sonicated in hexane and filtered under vacuum to afford the title product as a white powder, which was used in subsequent reactions without further purification (0.88 g, 87%).

^{1}H NMR (400 MHz $CDCl_3$) δ (ppm): 7.99 (d, *J* = 8.1 Hz, 2H, Ar*H*), 7.44 (d, *J* = 8.1 Hz, 2H, Ar*H*), 6.67 (d, *J* = 16.1 Hz, 1H, C*H*=CH), 6.48 (dt, *J* = 16.0, 5.4 Hz, 1H, C*H*=CH), 4.35 (dd, *J* = 13.3, 5.5 Hz, 2H, CH_2OH), 3.91 (d, *J* = 1.0 Hz, 3H, OCH_3). **^{13}C NMR** (101 MHz, $CDCl_3$): 166.9, 141.2, 131.4, 130.0, 129.8, 126.3, 63.4, 52.1. **HRMS**: (TOF MS ASAP+): Calculated for $C_{11}H_{13}O_3$ 193.0865 $[M+H]^+$. Found *m*/*z* 193.0865 $[M+H]^+$.

Methyl (*E*)-4-(3-bromoprop-1-en-1-yl)benzoate

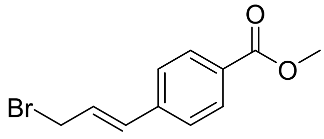

Methyl (*E*)-4-(2-hydroxyvinyl)benzoate (17.6 g, 91.6 mmol, 1 equiv.) and anh. diethyl ether (300 mL) were added to an oven-dried flask under argon. The flask was cooled to 0 °C before phosphorus tribromide (10.4 mL, 111 mmol, 1.2 equiv.) was added dropwise. The mixture was left to stir at 0 °C for 2h. Upon addition, a white solid precipitated out of solution rapidly. The mixture was then poured onto ice water and extracted with ethyl acetate. The organic layer was separated, washed with sodium bicarbonate, dried over $MgSO_4$ and filtered under vacuum before being concentrated *in vacuo.* The resulting solid was sonicated in hexane and filtered to afford the title product as a pearlescent white powder, which was used in subsequent reactions without further purification (14.4 g, 61%).

^{1}H NMR (600 MHz $CDCl_3$) δ (ppm): 8.03 – 7.97 (m, 2H, Ph*H*), 7.44 (d, *J* = 8.3 Hz, 2H, Ph*H*), 6.68 (d, *J* = 15.6 Hz, 1H, C*H*=CH), 6.50 (dt, *J* = 15.6, 7.7 Hz, 1H, C*H*=CH), 4.16 (dd, *J* = 7.7, 1.1 Hz, 2H, CH_2Br), 3.92 (s, 3H, OCH_3). **^{13}C NMR** (151 MHz, $CDCl_3$): 166.7, 140.2, 133.4, 130.0, 130.0, 130.0, 129.7, 127.8, 126.6, 52.1, 32.6. **HRMS**: (TOF MS ASAP+): Calculated for $C_{11}H_{12}O_2Br$ 255.0021 $[M+H]^+$. Found *m/z* 255.0027 $[M+H]^+$.

Methyl (*E*)-4-(3-(diethoxyphosphoryl)prop-1-en-1-yl)benzoate

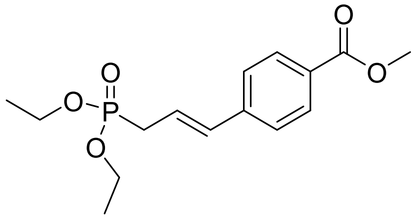

Methyl (*E*)-4-(3-bromoprop-1-en-1-yl)benzoate (14.3 g, 56.1 mmol, 1 equiv.) was heated to 160 °C with triethyl phosphite (11.5 mL, 67.3 mmol, 1.2 equiv.) for 20h. The resulting clear yellow mixture was then cooled to RT, dry loaded onto silica and purified by column chromatography (DCM:Acetone, 2:1) to afford the title product as a yellow-tinted clear oil (14 g, 80%).

^{1}H NMR (700 MHz CDCl$_3$) δ (ppm): 7.97 (d, *J* = 8.2 Hz, 2H, Ph*H*), 7.41 (d, *J* = 8.2 Hz, 2H, Ph*H*), 6.56 (dd, *J* = 15.7, 5.2 Hz, 1H, C*H*=CH), 6.29 (dd, *J* = 15.7, 7.6 Hz, 1H, C*H*=CH), 4.18 – 4.07 (m, 4H, CH_2CH$_3$), 3.90 (s, 3H, OCH_3), 2.84 – 2.74 (m, 2H, CH_2P), 1.32 (t, *J* = 7.1 Hz, 6H, CH$_2$CH_3). **^{13}C NMR** (176 MHz, CDCl$_3$): 166.8, 141.2 , 133.9, 130.0, 129.1, 126.1, 121.9, 62.2, 52.1, 31.7, 16.5. **HRMS**: (TOF MS ASAP+): Calculated for $C_{15}H_{22}O_5P$ 313.1205 [M+H]$^+$. Found *m/z* 313.1207 [M+H]$^+$.

(*E*)-3-(3,5-Di-tert-butylphenyl)acrylaldehyde

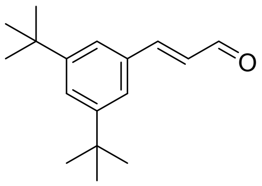

Procedure from[5]

3,5-ditertbutylbenzaldehyde (7.02 g, 32.2 mmol, 1.5 equiv.), (triphenylphosphoranylidene)acetaldehyde (6.53 g, 21.5 mmol, 1.0 equiv.) and anh. toluene (200 mL) were added to an oven-dried flask equipped with a reflux condenser and under argon. The mixture was stirred at 130 ^{o}C for 24h, before being concentrated *in vacuo,* dry loaded onto silica and purified via column chromatography (Hexane: DCM, 1:1). Mixed fractions containing product and unreacted starting materials were combined, concentrated and re-purified. The fractions containing the product were concentrated *in vacuo*, to afford the title product as a viscous yellow oil (1.59 g, 30%).

^{1}H NMR (700 MHz CDCl$_3$) δ (ppm): 9.71 (d, *J* = 7.7 Hz, 1H, CO*H*), 7.56 – 7.49 (m, 2H, Ar*H*), 7.41 (d, *J* = 1.8 Hz, 2H, Ar*H*), 6.75 (dd, *J* = 15.9, 7.7 Hz, 1H, Ar*H*), 1.35 (s, 18H, C*H*$_3$).**^{13}C NMR** (176 MHz, CDCl$_3$): 194.0, 154.3, 151.8, 133.4, 128.2, 125.9, 122.9, 34.9, 31.3. **HRMS:** (TOF MS ASAP+): Calculated for $C_{17}H_{25}O$: 245.1905 [M+H]+. Found m/z 245.1911 [M+H]+

Methyl 4-((1*E*,3*E*,5*E*)-6-(3,5-di-tert-butylphenyl)hexa-1,3,5-trien-1-yl)benzoate

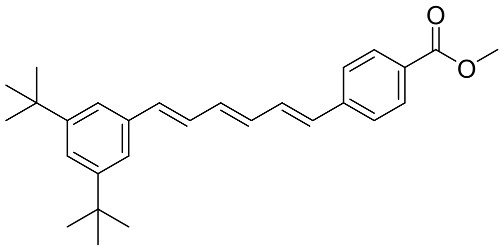

Methyl (*E*)-4-(3-(diethoxyphosphoryl)prop-1-en-1-yl)benzoate (1.28 g, 4.10 mmol, 1.0 equiv.) and anh. THF (18 mL) were added to an oven-dried flask under argon. Then, sodium hydride (0.055 g, 2.29 mmol, 0.56 equiv.) was added, followed by more anh.THF (4.8 mL). The mixture was stirred at RT for 30 mins to give a dark red solution. Then, a pre-dissolved solution of (*E*)-3-(3,5-di-tert-butylphenyl)acrylaldehyde (1.30 g, 5.32 mmol, 1.30 equiv.) in anh. THF (18 mL) was added slowly. The resulting mixture was stirred for another 30 mins at RT. A second dose of sodium hydride (0.059 g, 2.46 mmol, 0.60 equiv.) was added, followed by more anh. THF (4.8 mL). The resulting mixture was left to stir at RT for 24h. The resulting solution was hydrolysed with water and then extracted with diethyl ether (1M HCl solution can be added to help separate layers). The organic phases were separated and dried over $MgSO_4$, filtered through cotton wool, and concentrated *in vacuo*. The crude product was dry loaded onto silica and purified *via* column chromatography (Hexane: DCM (3:2). Fractions containing the product were combined and concentrated *in vacuo*, before being recrystallised from DCM:MeOH to afford the title product as a yellow crystals (with a small isomeric impurities) (0.22 g, 14%).

^{1}H NMR (700 MHz $CDCl_3$) 7.98 (d, *J* = 7.9 Hz, 2H, Ar*H*), 7.46 (d, *J* = 8.0 Hz, 2H, Ar*H*), 7.34 (s, 1H, Ar*H*), 7.28 (s, 2H, Ar*H*), 6.99 (dd, *J* = 15.7, 10.5 Hz, 1H,), 6.89 (dd, *J* = 15.2, 10.9 Hz, 1H, C*H*=CH), 6.68 (d, *J* = 15.4 Hz, 1H, C*H*=CH), 6.63 – 6.51 (m, 3H, C*H*=CH, and Ar*H*), 3.91 (s, 3H, OCH_3), 1.35 (d, *J* = 2.3 Hz, 18H, CH_3). **^{13}C NMR** (176 MHz, $CDCl_3$): 166.9, 151.1, 142.0, 136.4, 135.6, 135.0, 132.5, 131.9, 131.0, 130.0, 128.6, 128.2, 126.1, 122.4, 120.9, 52.1, 34.9, 31.4. **HRMS**: (TOF MS ASAP+): Calculated for $C_{28}H_{34}O_2$: 402.2559 $[M]^+$. Found *m*/*z* 402.2555 $[M]^+$.

4-((1*E*,3*E*,5*E*)-6-(3,5-Di-tert-butylphenyl)hexa-1,3,5-trien-1-yl)benzoic acid

DTB-DPH-CA

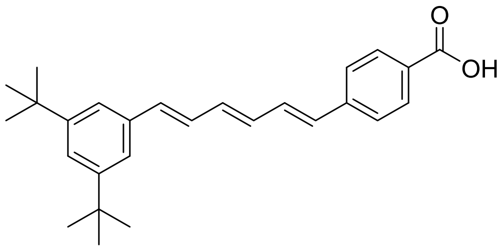

Procedure amended from[6]

Methyl 4-((1*E*,3*E*,5*E*)-6-(3,5-di-tert-butylphenyl)hexa-1,3,5-trien-1-yl)benzoate (0.20 g, 0.497 mmol, 1.0 equiv.) and anh. methanol (6.6 mL) were added to an oven-dried flask under argon. The flask was warmed to 35 ^{o}C before potassium hydroxide (0.28 g, 5.06 mmol, 10 equiv.) was added. The mixture was heated to reflux for 24h. Ice water was then added, causing a yellow solid to initially precipitate out of solution but upon warming to RT, dissolved. Therefore, the solution was acidified with 6N HCl and extracted with diethyl ether. The organic phases were separated and dried over $MgSO_4$, filtered through cotton wool, and concentrated *in vacuo*. The crude product was sonicated in methanol and filtered to afford the title product as a shiny yellow flocculent powder (0.092 g, 48%).

^{1}H NMR (400 MHz $CDCl_3$) δ (ppm): 8.08 – 8.00 (m, 2H, Ar*H*), 7.54 – 7.45 (m, 2H, Ar*H*), 7.36 – 7.27 (m, 3H Ar*H*), 7.02 (dd, J = 15.5, 10.0 Hz, 1H, C*H*=CH), 6.90 (dd, J = 15.5, 9.9 Hz, 1H, C*H*=CH), 6.74 – 6.50 (m, 4H, C*H*=CH), 1.64 (brs, 1H, O*H*), 1.35 (s, 18H, CH_3). **^{13}C NMR** (176 MHz, $CDCl_3$): 170.9, 151.1, 142.9, 136.3, 135.9, 135.2, 132.4, 132.3, 130.8, 130.7, 128.2, 127.6, 126.2, 122.5, 120.9, 34.9, 31.4. **HRMS**: (TOF MS ASAP+): Calculated for $C_{27}H_{32}O_2$ 388.2402 $[M]^+$. Found *m*/*z* 388.2406 $[M]^+$.

S2 - 1H NMR and 13C NMR Spectra

Tetraethyl but-2-ene-1,4-diyl(*E*)-bis(phosphonate)

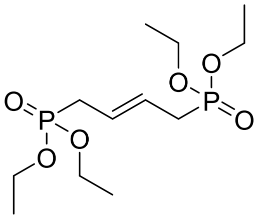

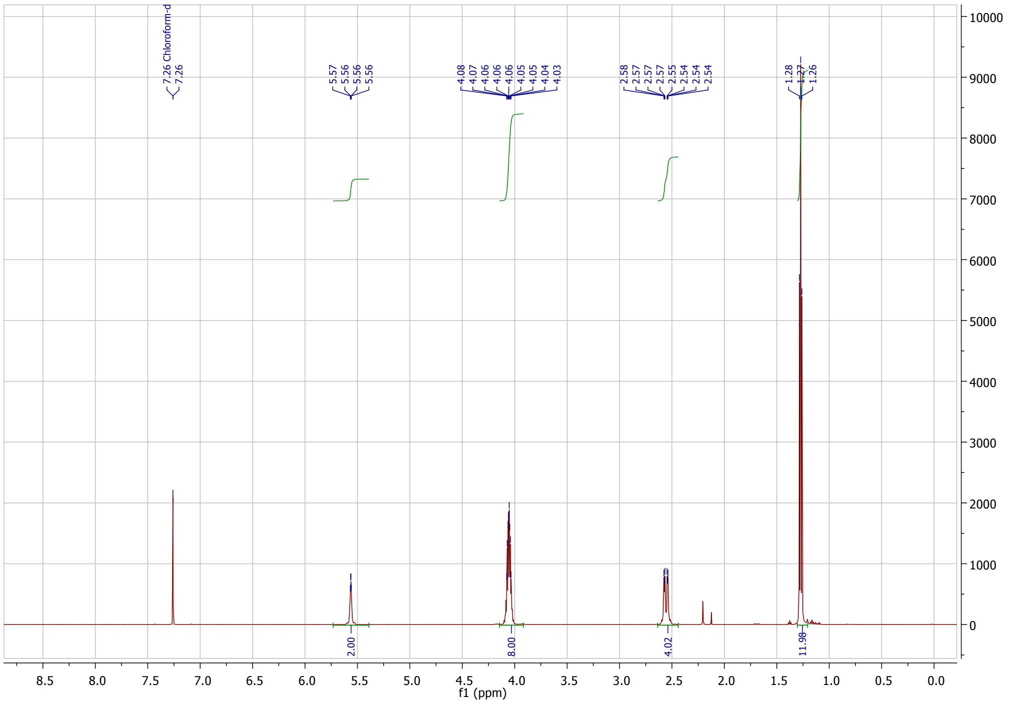

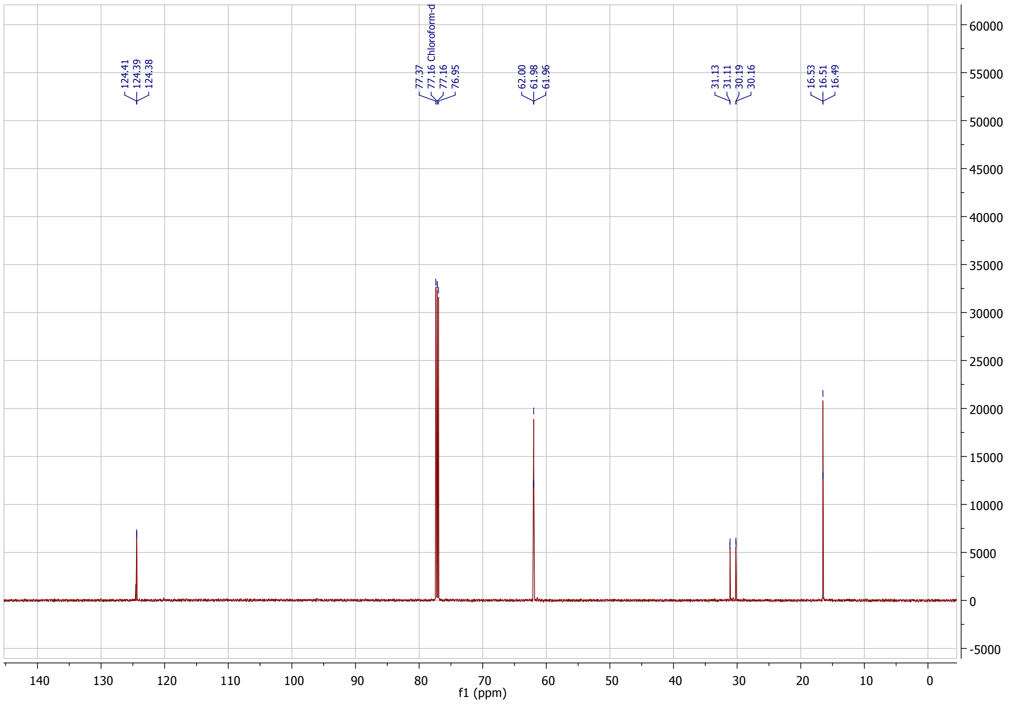

(1*E*,3*E*,5*E*)-1,6-Bis(3,5-di-tert-butylphenyl)hexa-1,3,5-triene

DPH1-(TTB)

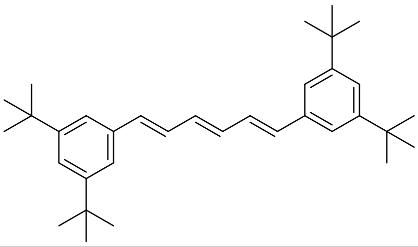

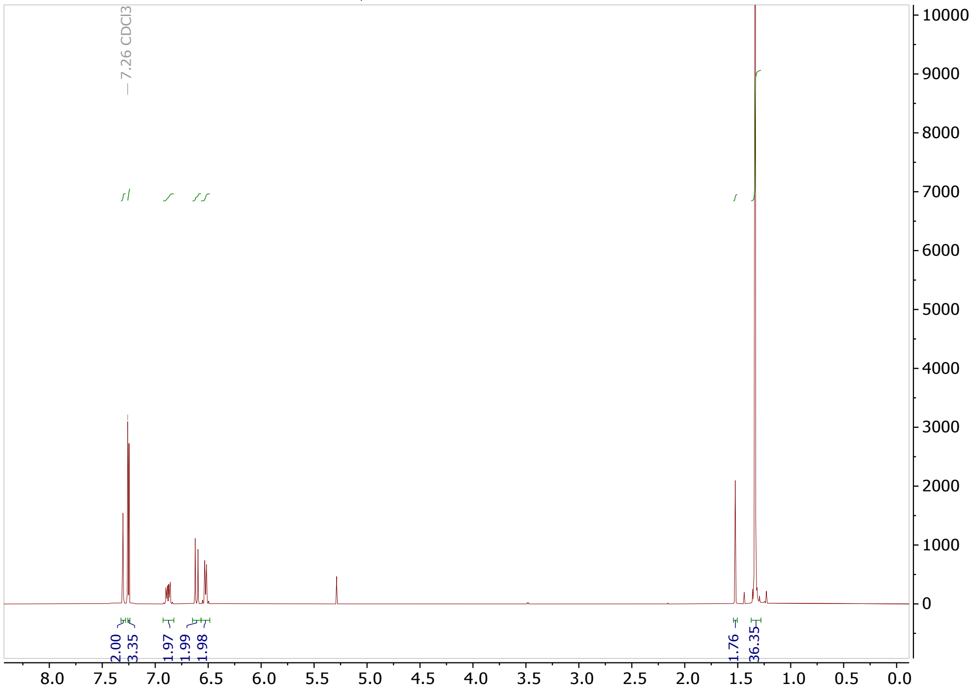

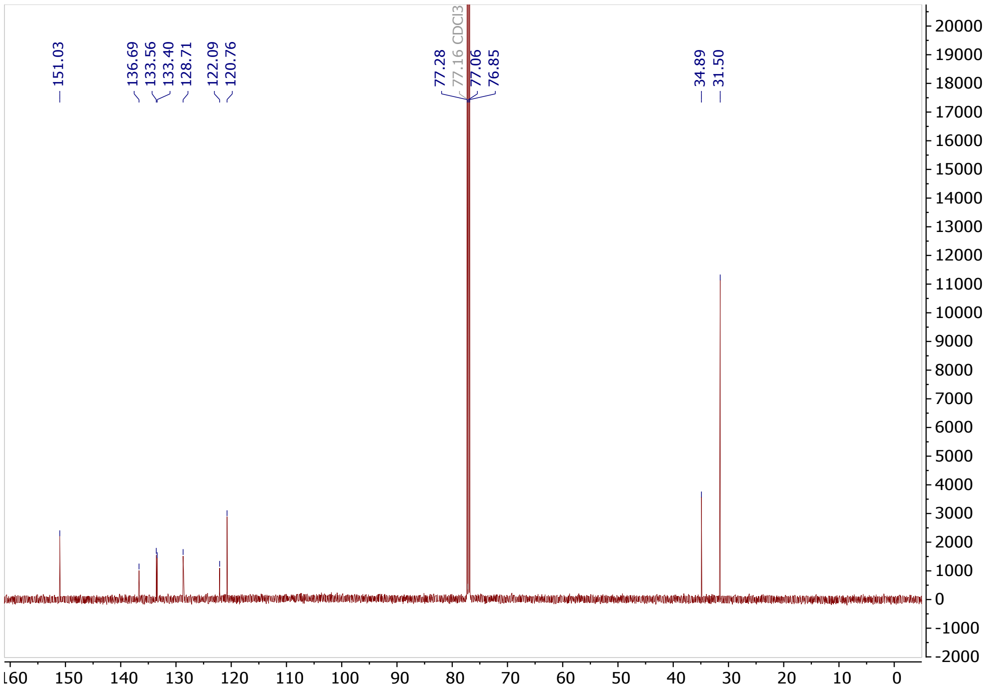

(1*E*,3*E*,5*E*)-1,6-Bis(3,5-dimethoxyphenyl)hexa-1,3,5-triene

DPH2-(TM)

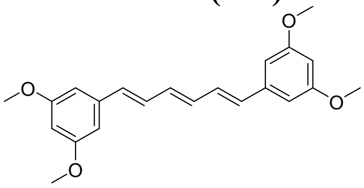

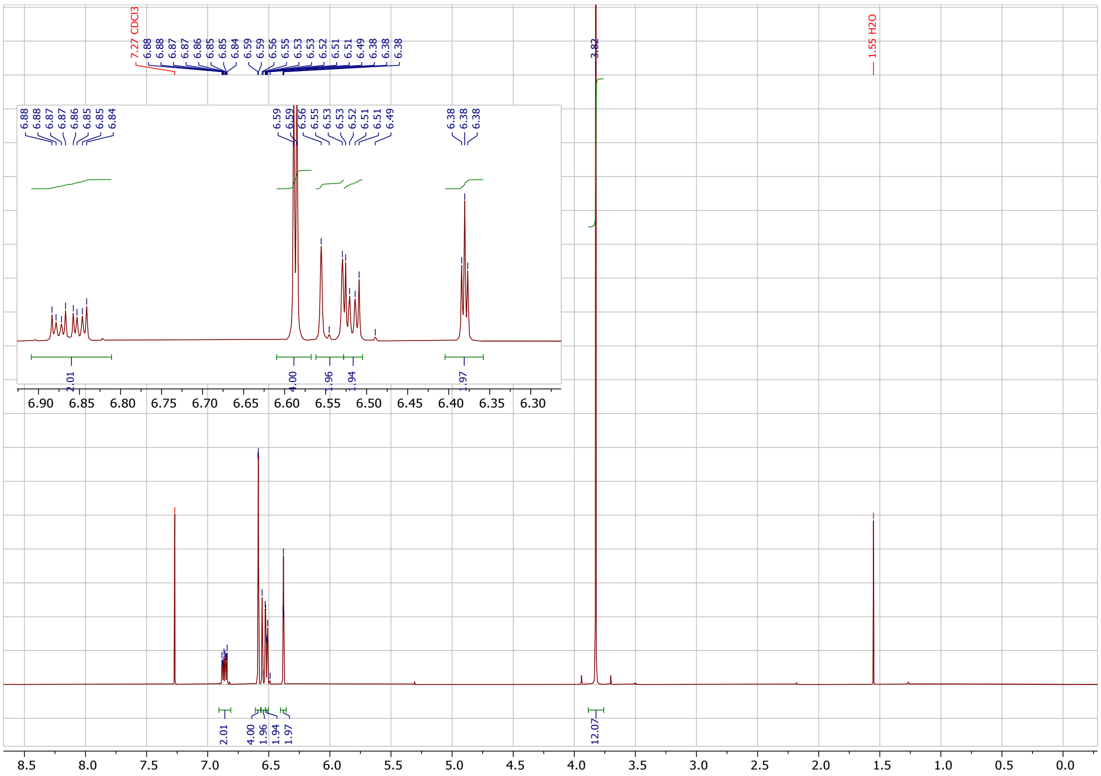

7.27 CDCl3
6.88
6.88
6.87
6.87
6.86
6.85
6.85
6.84
6.59
6.59
6.56
6.55
6.53
6.53
6.52
6.51
6.51
6.49
6.38
6.38
6.38
3.82
1.55 H2O
2.01
4.00
1.96
1.94
1.97
12.07

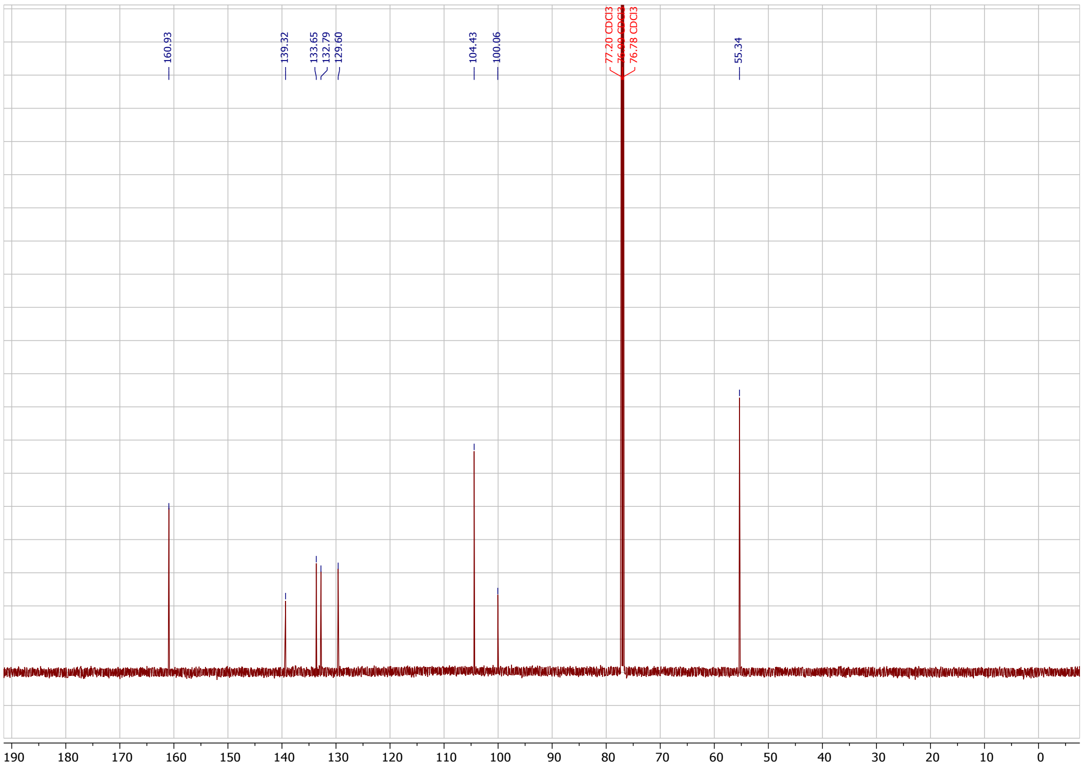

160.93
139.32
133.65
132.79
129.60
104.43
100.06
77.20 CDCl3
76.78 CDCl3
55.34

3,5-Bis(isopentyloxy)benzaldehyde

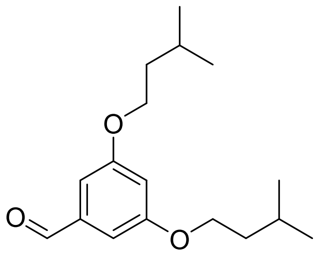

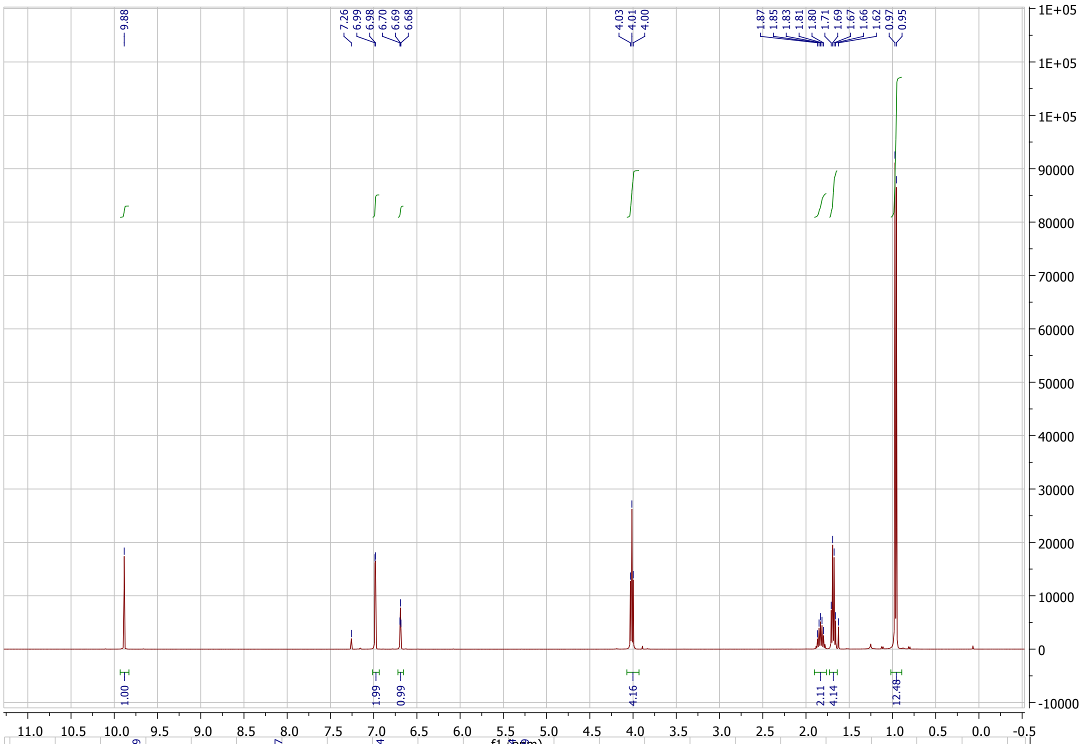

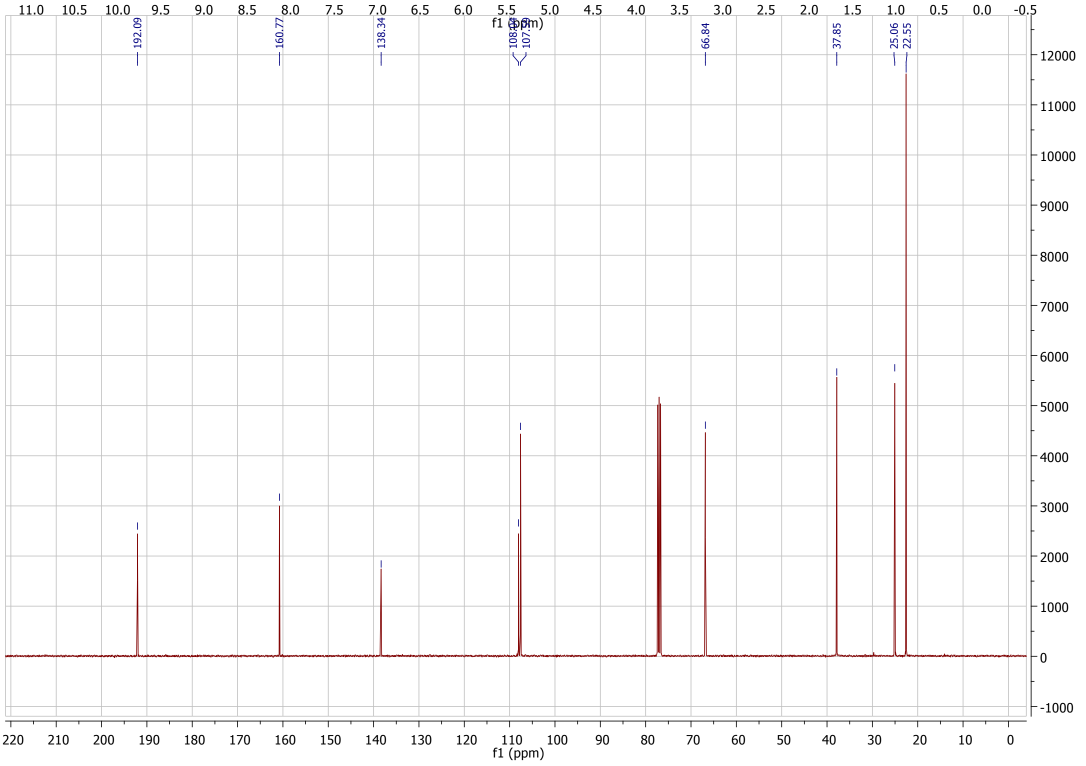

(1*E*,3*E*,5*E*)-1,6-Bis(3,5-bis(isopentyloxy)phenyl)hexa-1,3,5-triene

DPH3-(TIPO)

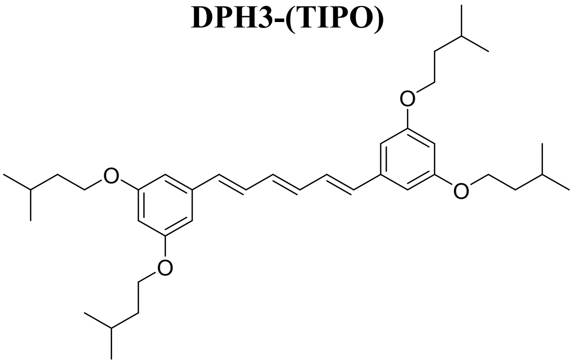

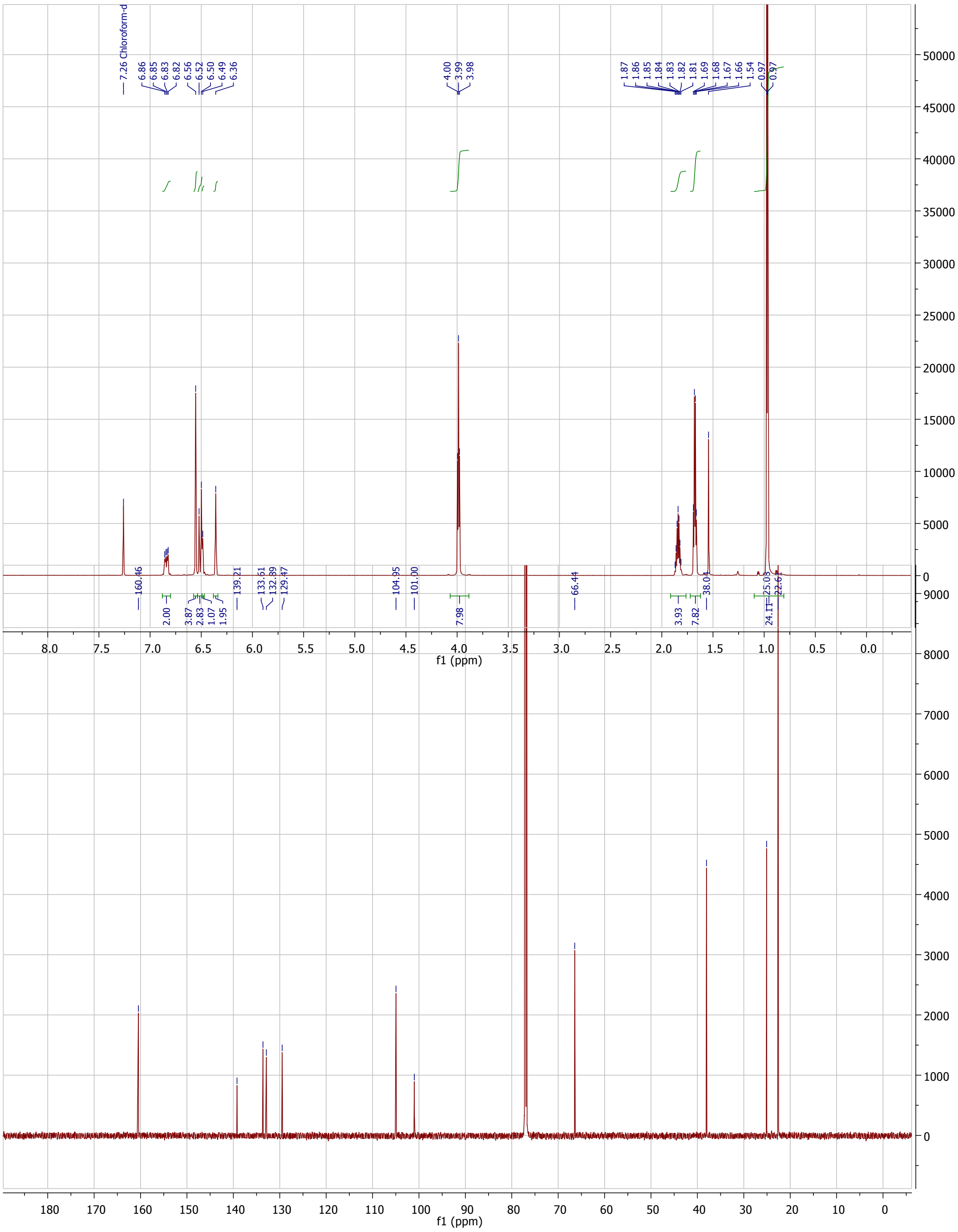

4-((Triisopropylsilyl)ethynyl)benzaldehyde

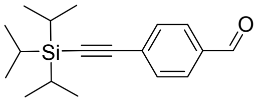

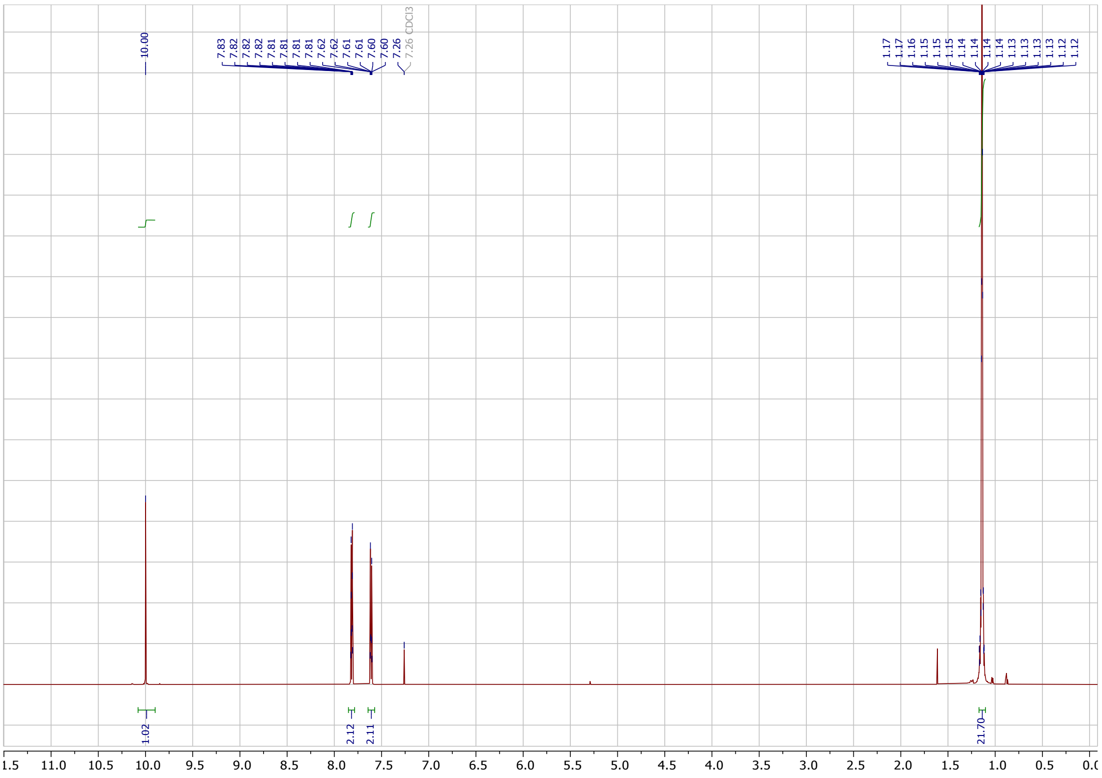

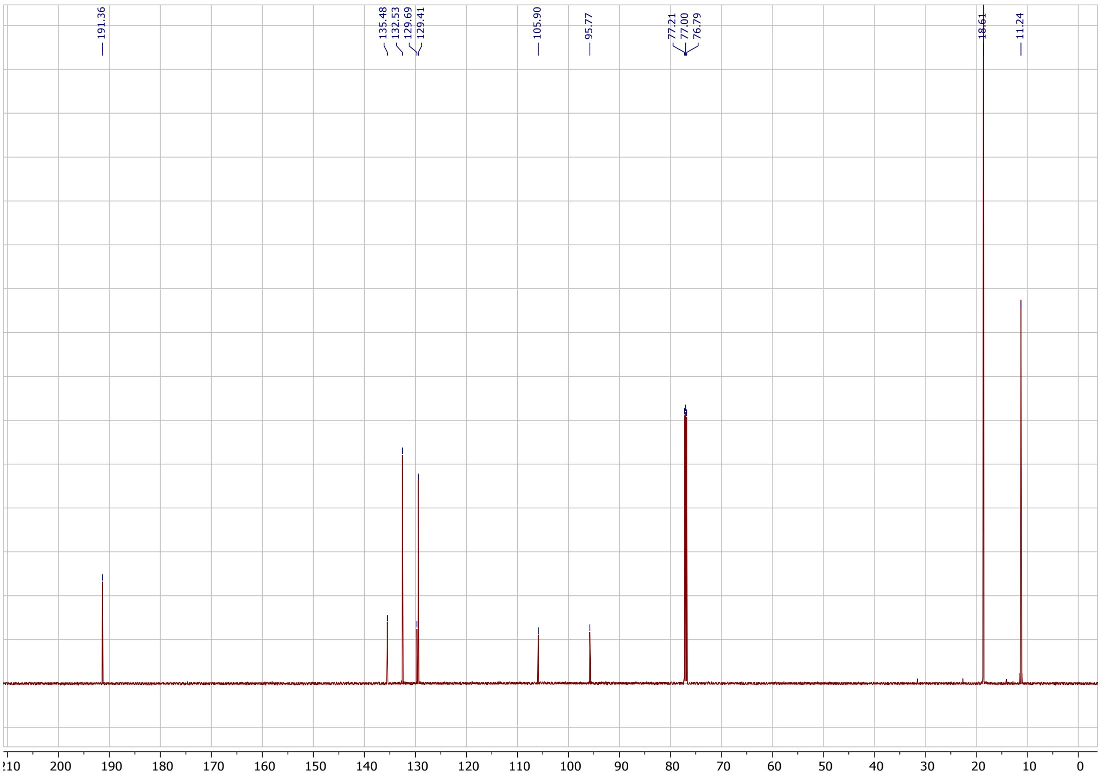

(1*E*,3*E*,5*E*)-1,6-Bis(4-((triisopropylsilyl)ethynyl)phenyl)hexa-1,3,5-triene

DPH4-(BATIPS)

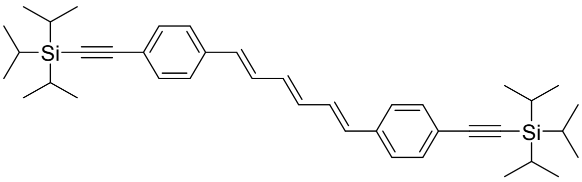

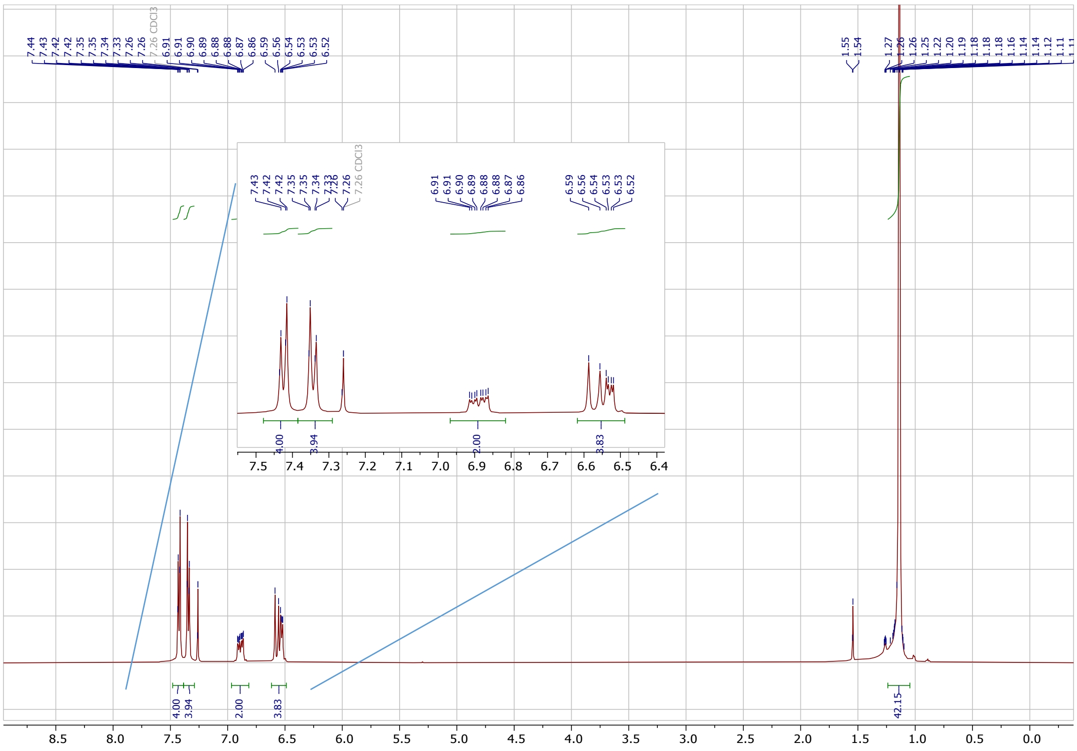

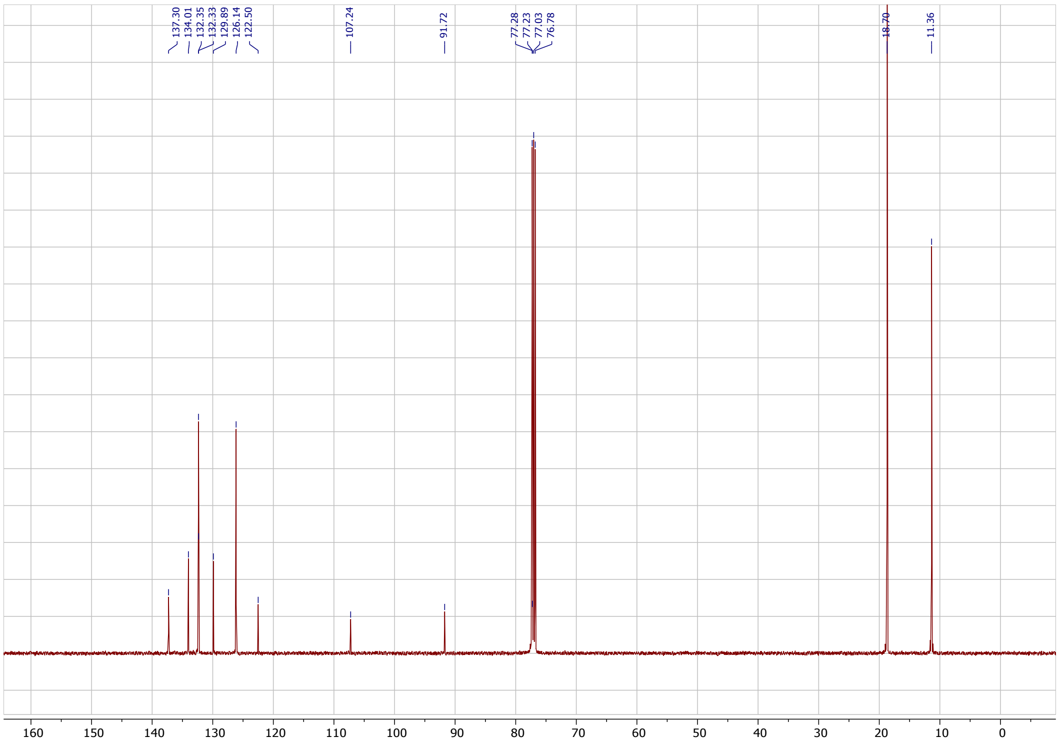

Methyl (*E*)-4-(3-oxoprop-1-en-1-yl)benzoate

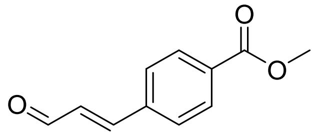

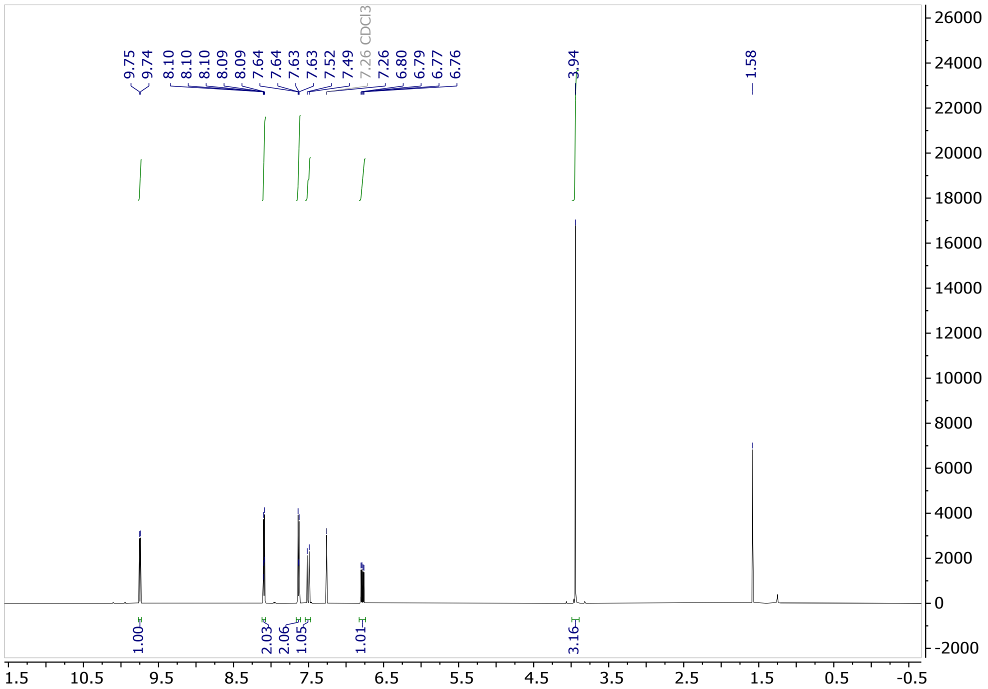

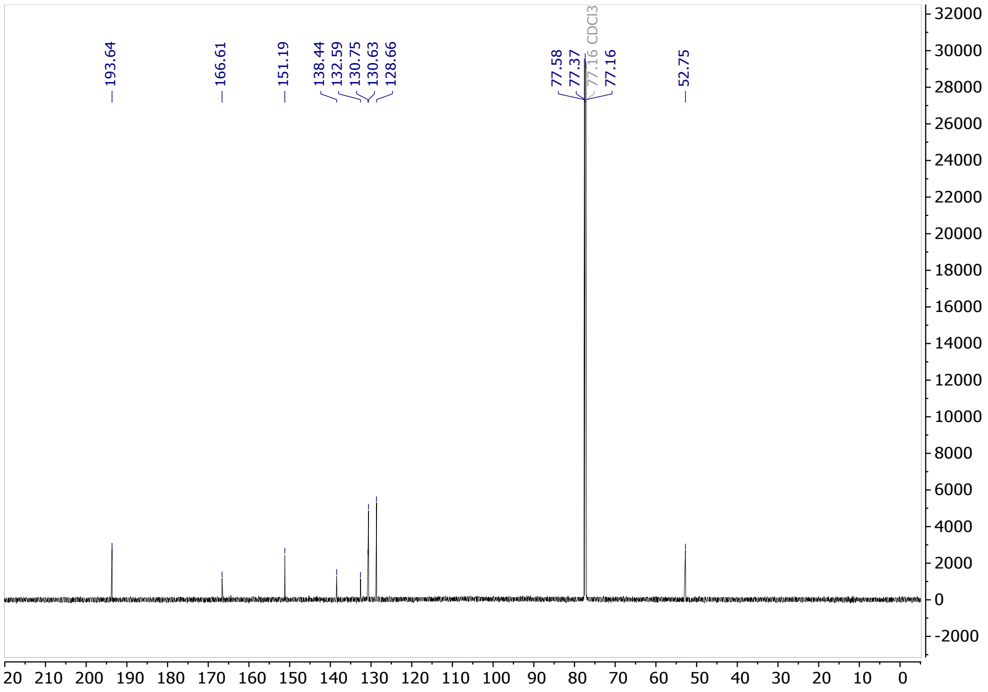

Methyl (*E*)-4-(2-hydroxyvinyl)benzoate

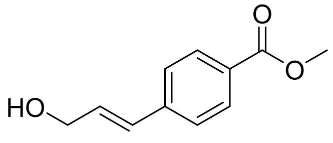

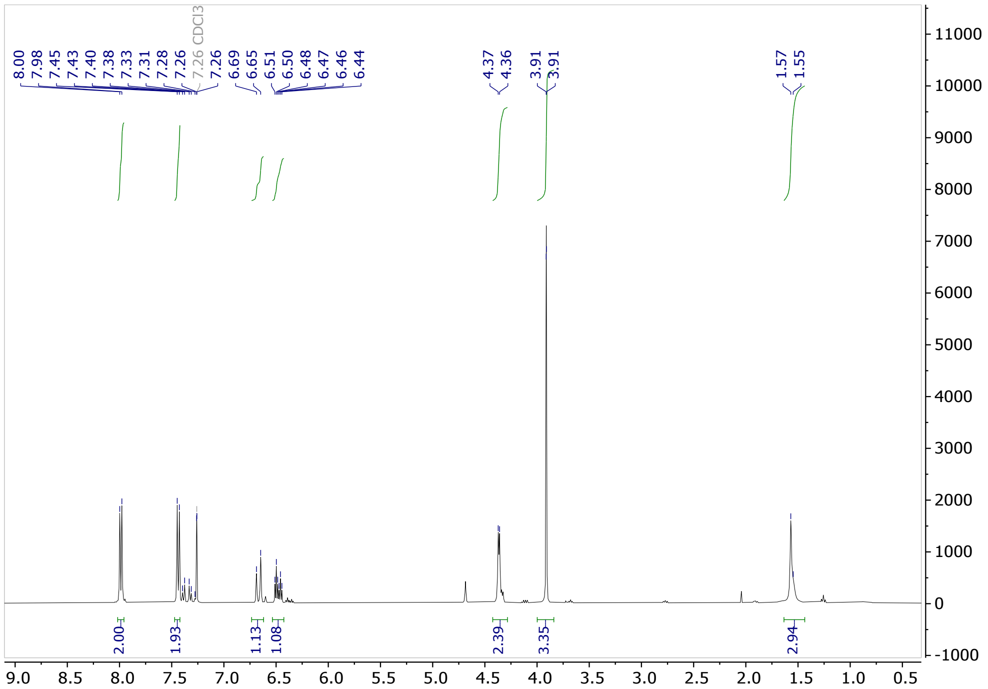

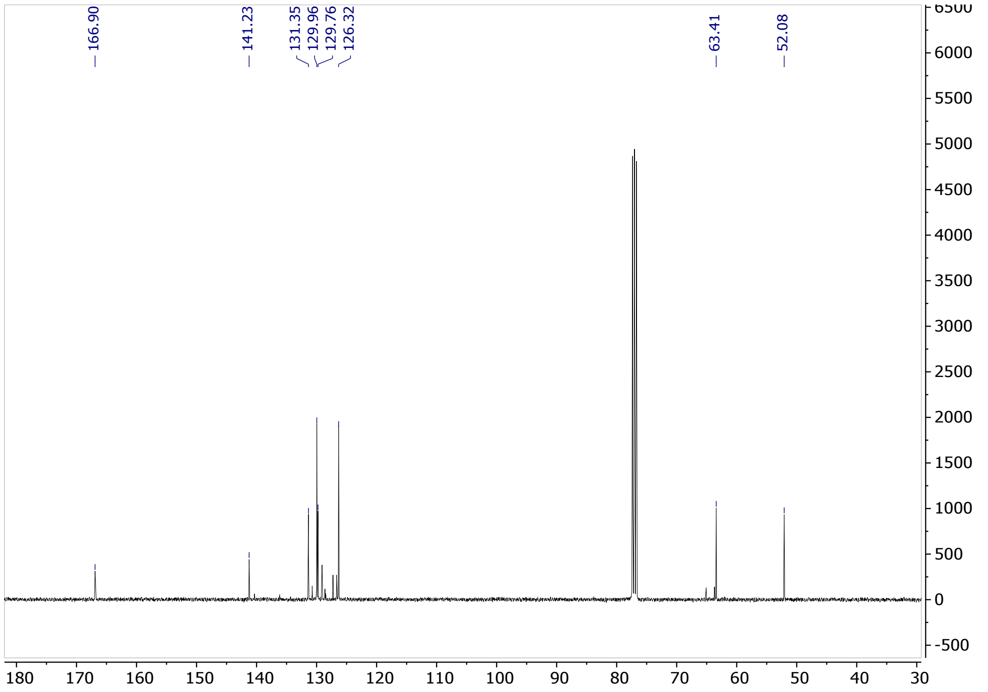

Methyl (*E*)-4-(3-bromoprop-1-en-1-yl)benzoate

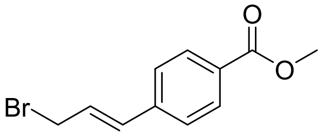

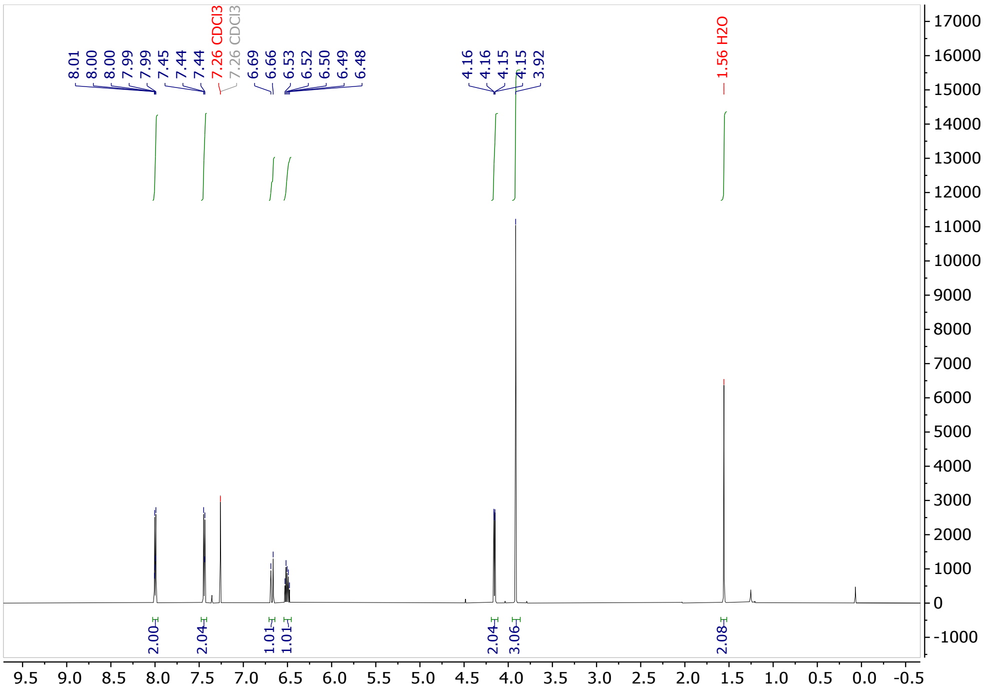

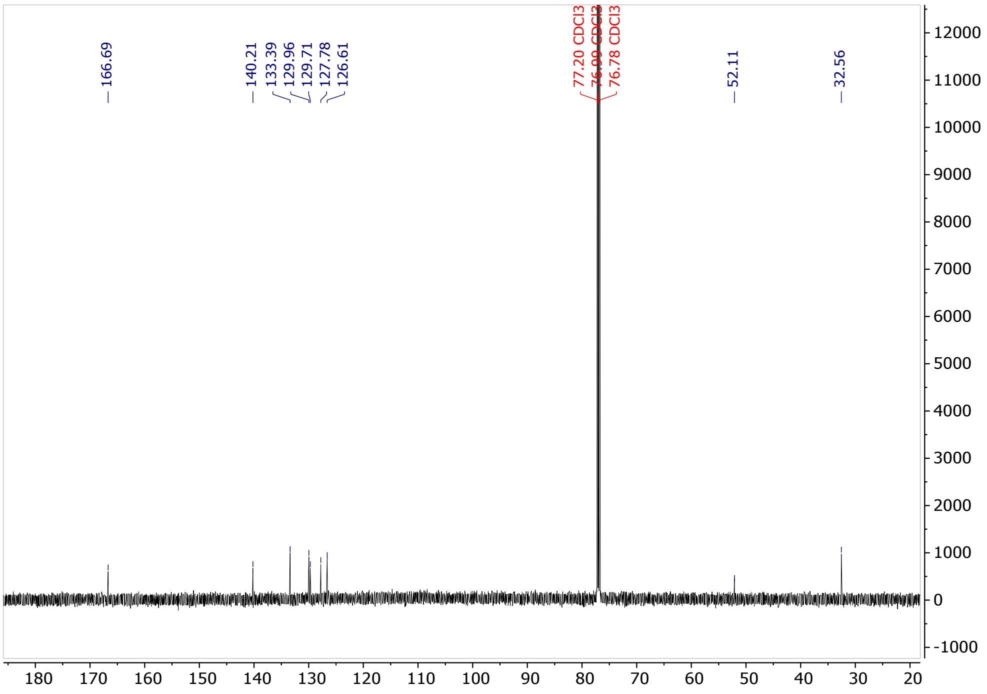

Methyl (*E*)-4-(3-(diethoxyphosphoryl)prop-1-en-1-yl)benzoate

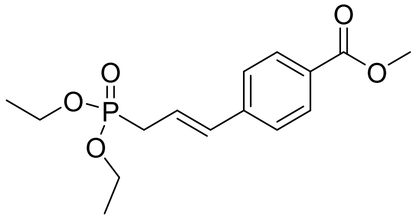

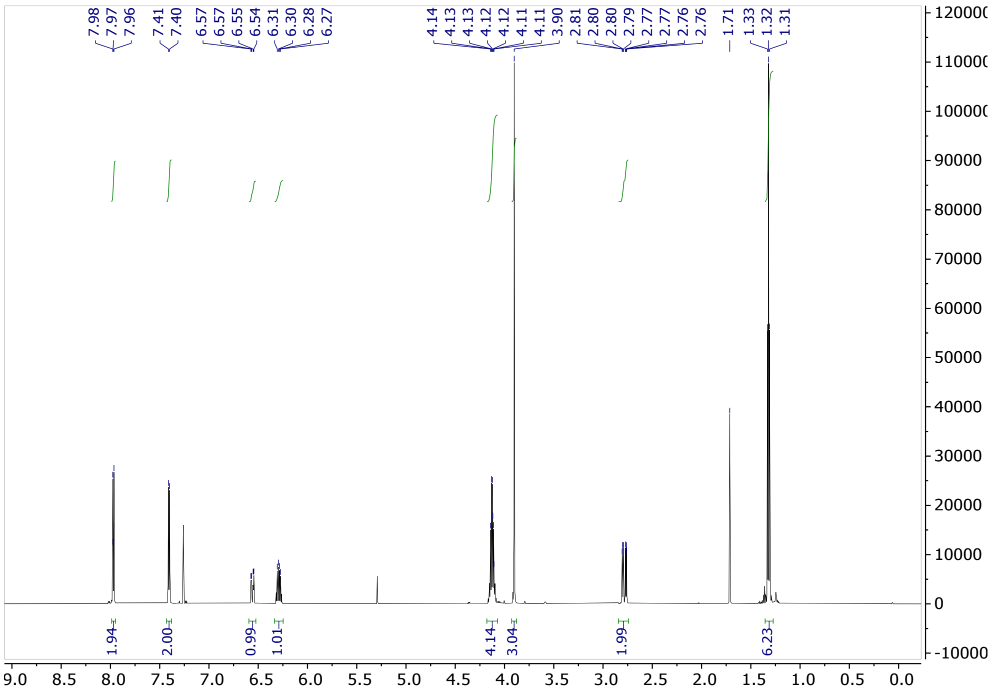

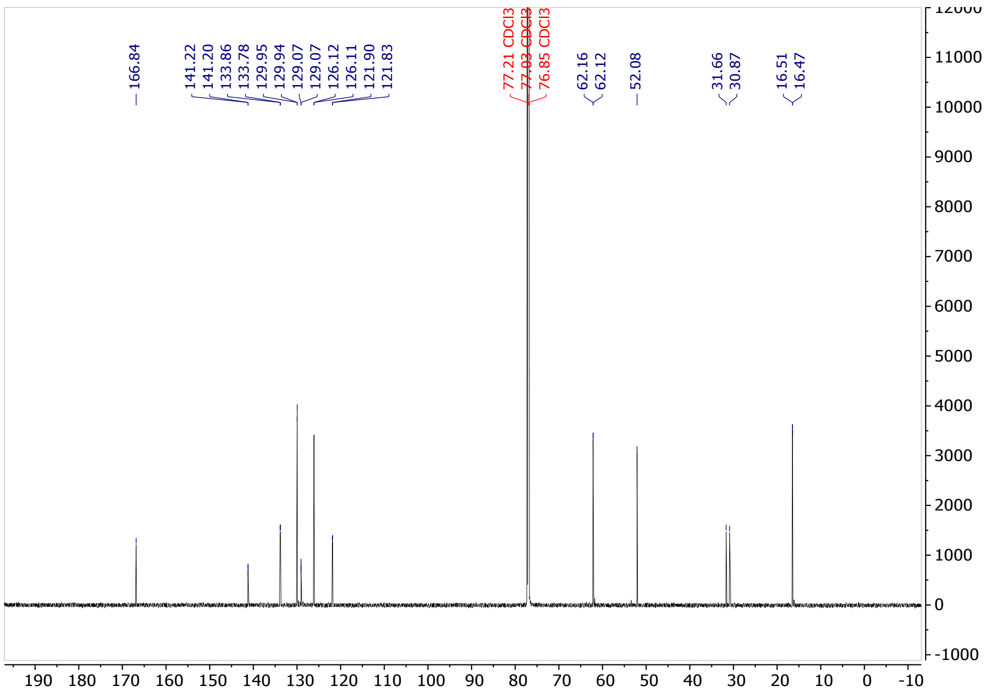

(*E*)-3-(3,5-Di-tert-butylphenyl)acrylaldehyde

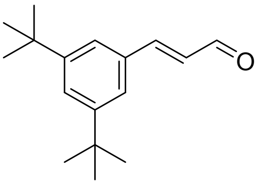

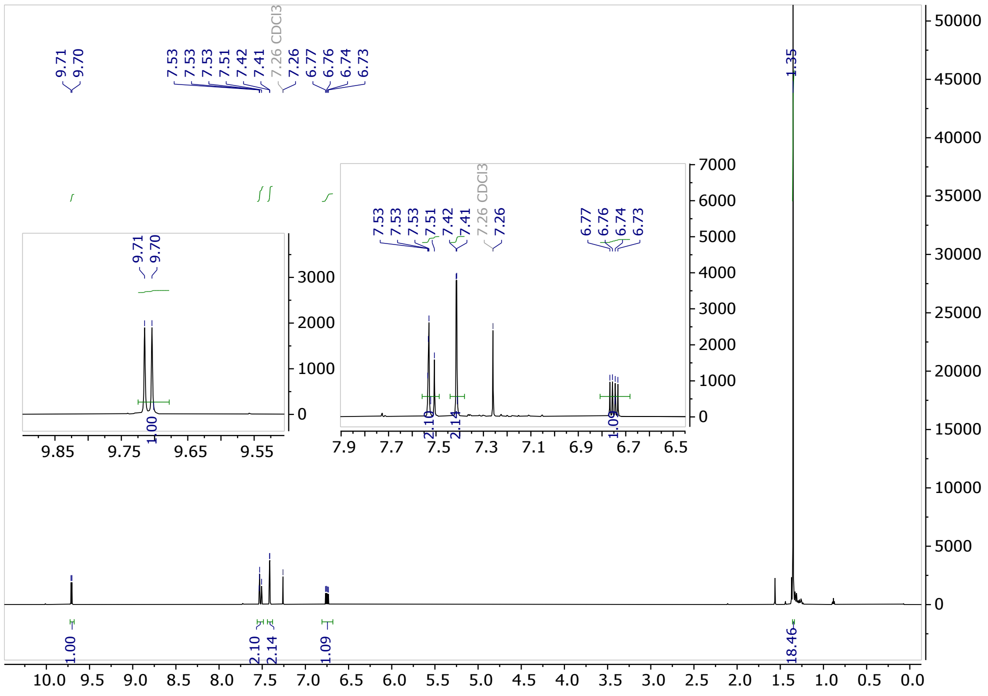

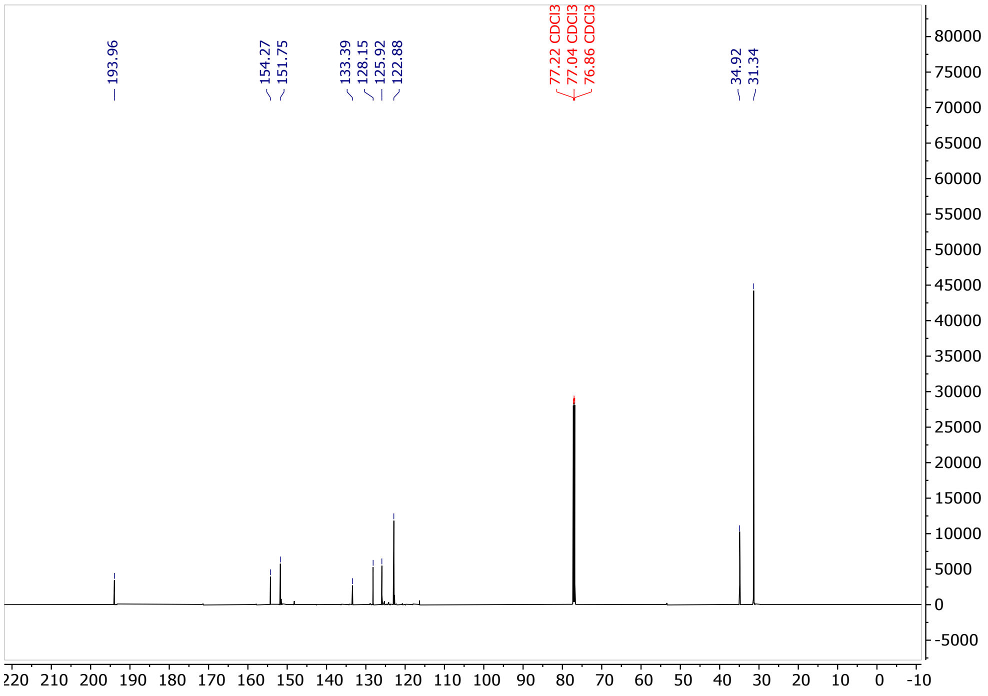

Methyl 4-((1*E*,3*E*,5*E*)-6-(3,5-di-tert-butylphenyl)hexa-1,3,5-trien-1-yl)benzoate

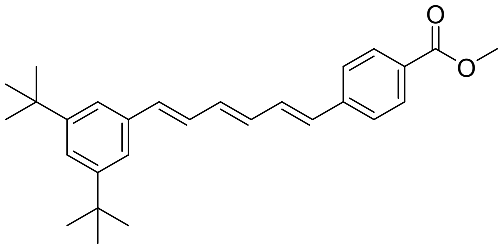

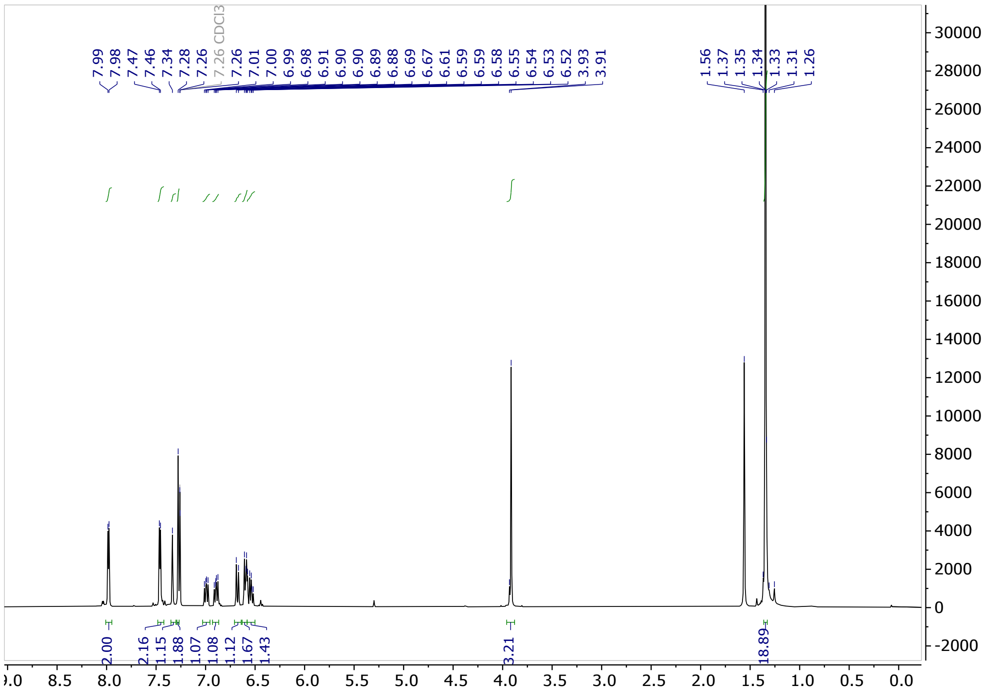

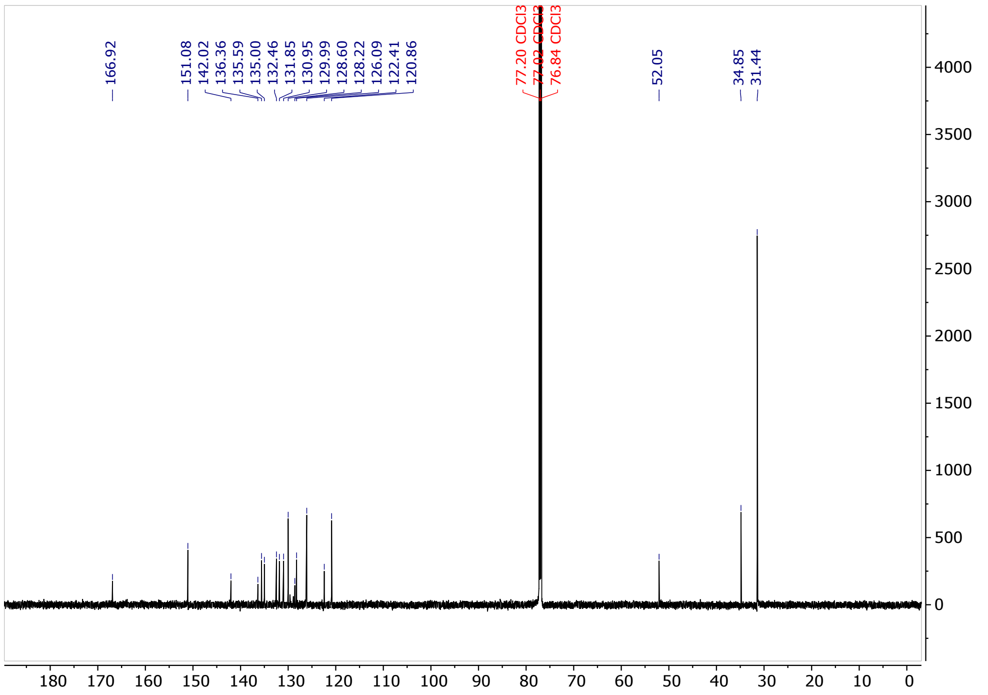

4-((1*E*,3*E*,5*E*)-6-(3,5-Di-tert-butylphenyl)hexa-1,3,5-trien-1-yl)benzoic acid

DTB-DPH-CA

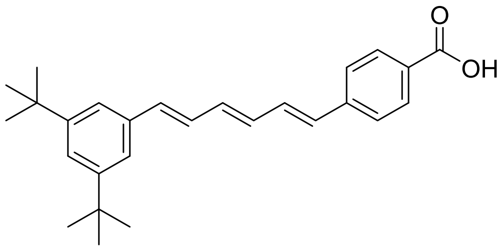

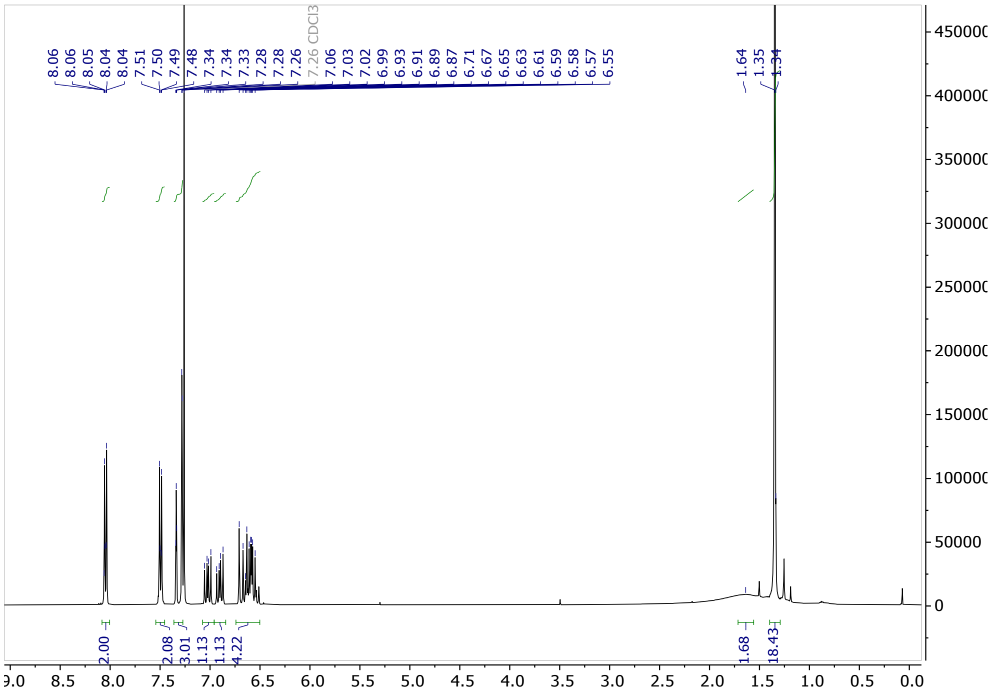

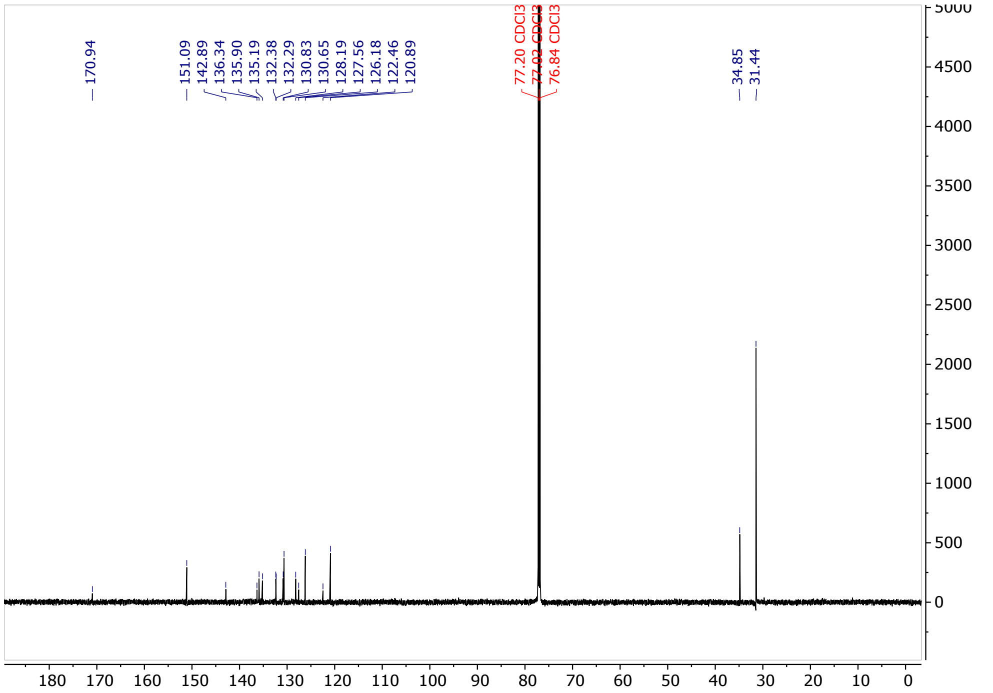

S3: Single Crystal Structures of the DPH Derivatives

Table S1: *Crystallographic information for single crystal structures of DPH4-(BATIPS), DPH3-(TIPO), DPH2-(TM) and DPH1-(TTB).*

	**DPH4-(BATIPS)**	**DPH3-(TIPO)**	**DPH2-(TM)**	**DPH1-(TTB)**
CCDC number	2469741	2469743	2469744	2469742
Cambridge data number	HB_K1_0015	HB_K1_0005	HB_K1_0013	HB_K1_0011
Chemical formula	$C_{40}H_{56}Si_2$	$C_{38}H_{56}O_4$	$C_{22}H_{24}O_4$	$C_{34}H_{48}$
Formula weight	593.02	576.82	352.41	456.72
Temperature / K	180(2)	180(2)	180(2)	180(2)
Crystal system	triclinic	monoclinic	monoclinic	monoclinic
Space group	P –1	P 2_1/n	P 2_1/c	P 2_1/c
a / Å	7.6026(4)	6.0878(3)	14.6197(7)	13.7983(6)
b / Å	7.9686(5)	18.4230(10)	9.5707(3)	10.1762(4)
c / Å	16.0548(12)	16.1857(11)	13.6916(5)	10.8134(3)
alpha / °	89.365(2)	90	90	90
beta / °	82.472(3)	98.216(2)	105.241(2)	100.9137(13)
gamma / °	74.550(4)	90	90	90
Unit-cell volume / Å^3	929.12(10)	1796.68(18)	1848.36(13)	1490.89(10)
Z	1	2	4	2
Calc. density / g cm^{-3}	1.060	1.066	1.266	1.017
F(000)	324	632	752	504
Radiation type	Mo Ka	Mo Ka	Mo Ka	Mo Ka
Absorption coeff / mm^{-1}	0.120	0.067	0.086	0.056
Crystal size / mm^3	0.40 x 0.14 x 0.04	0.50 x 0.05 x 0.05	0.50 x 0.50 x 0.10	0.45 x 0.25 x 0.25
2-Theta range / °	7.31-50.77	7.11-50.79	7.18-50.05	7.23-50.05
Completeness to max 2q	0.954	0.964	0.995	0.995
No. of refl measured	7125	10566	10036	8038
No. of independent refl	3269	3204	3253	2627
R(int)	0.0782	0.1243	0.0618	0.0389
No. params / restraints	196 / 0	233 / 56	239 / 0	160 / 0
Final R1 value (I > 2s(I))	0.0594	0.1015	0.0447	0.0396
Final wR(F^2) value (all data)	0.1372	0.1892	0.1064	0.1103
Goodness-of-fit on F^2	1.036	1.095	0.915	1.010
Largest diff peak & hole / e Å^{-3}	0.215, -0.212	0.339, -0.190	0.163, -0.169	0.133, -0.137

S3.1 - Single-crystal X-ray diffraction

Single-crystal X-ray data were collected at 180(2) K on a Nonius KappaCCD diffractometer, using graphite-monochromated MoKa radiation (l = 0.7107 Å). Structures were solved using SHELXT (Sheldrick, 2015) and refined using SHELXL (Sheldrick, 2015). Structure solution and refinement was mostly straightforward, with all non-H atoms refined anisotropically and H atoms placed in idealised positions and refined as riding. DPH3-(TIPO) crystallised as thin needles. Diffraction was quite weak, with I/s(I) dropping below 3.0 around 1.00 Å resolution. Data were integrated to 0.84 Å, but R(int) and R1 are relatively higher than the other structures. DPH3-(TIPO) also showed some disorder in one —$CH_2CH_2{}^iPr$ group, which was modelled as two components.

SHELXT: G. M. Sheldrick, Acta Cryst. Sect. A, 2015, 71, 3–8.

SHELXL: G. M. Sheldrick, Acta Cryst. Sect. C, 2015, 71, 3–8.

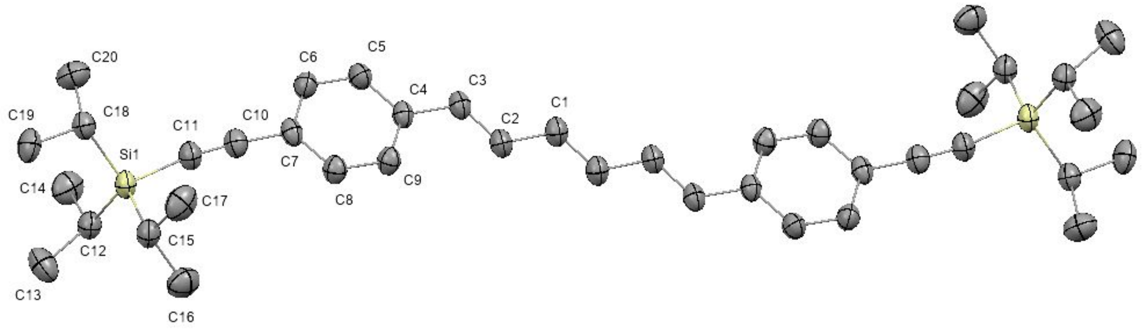

Figure S1: *Molecular structure of* ***DPH4-(BATIPS)*** *with displacement ellipsoids at 50% probability. H atoms are omitted. The molecule is situated on an inversion centre in space group P–1.*

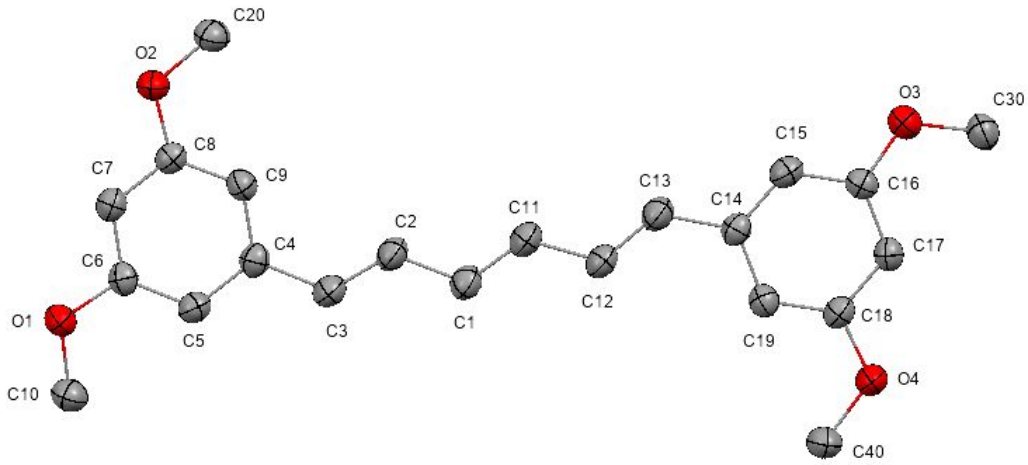

Figure S2: *Molecular structure of* ***DPH2-(TM)*** *with displacement ellipsoids at 50% probability. H atoms are omitted. The molecule is situated on a general position in space group $P2_1/c$: inversion symmetry is broken by the orientations of the OMe groups in the crystal.*

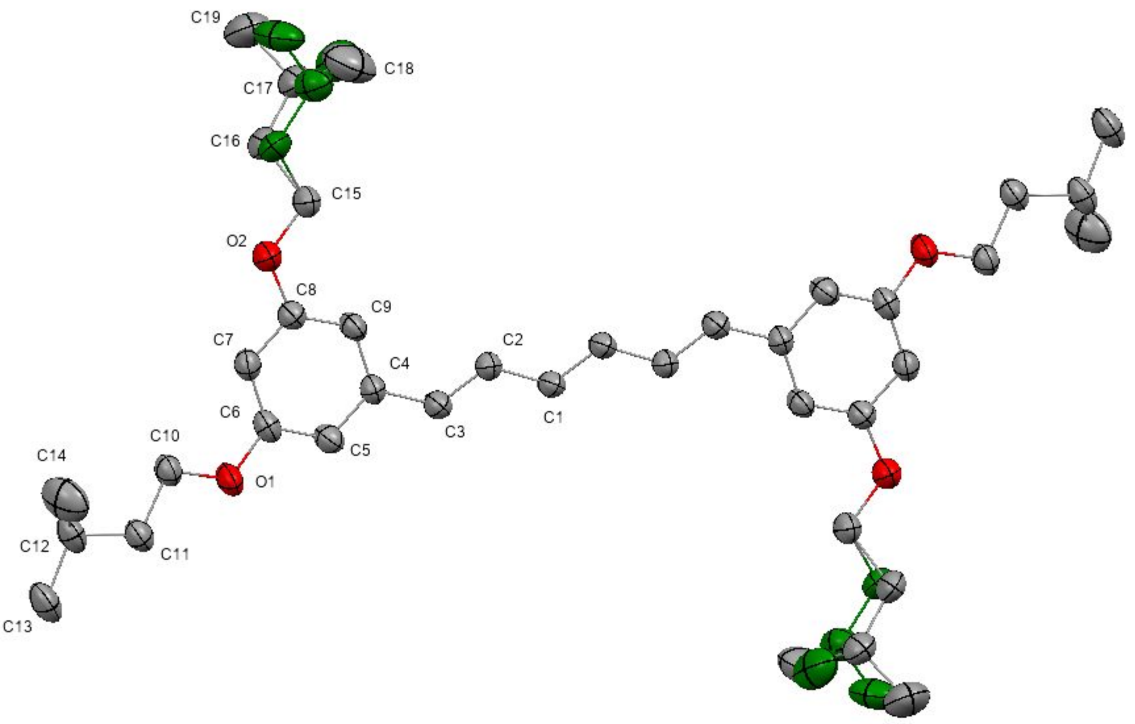

Figure S3: *Molecular structure of* ***DPH3-TIPO*** *with displacement ellipsoids at 50% probability. H atoms are omitted. The molecule is situated on an inversion centre in space group* $P2_1/n$*. A second disorder component in the —*$CH_2CH_2{}^iPr$ *group is coloured green.*

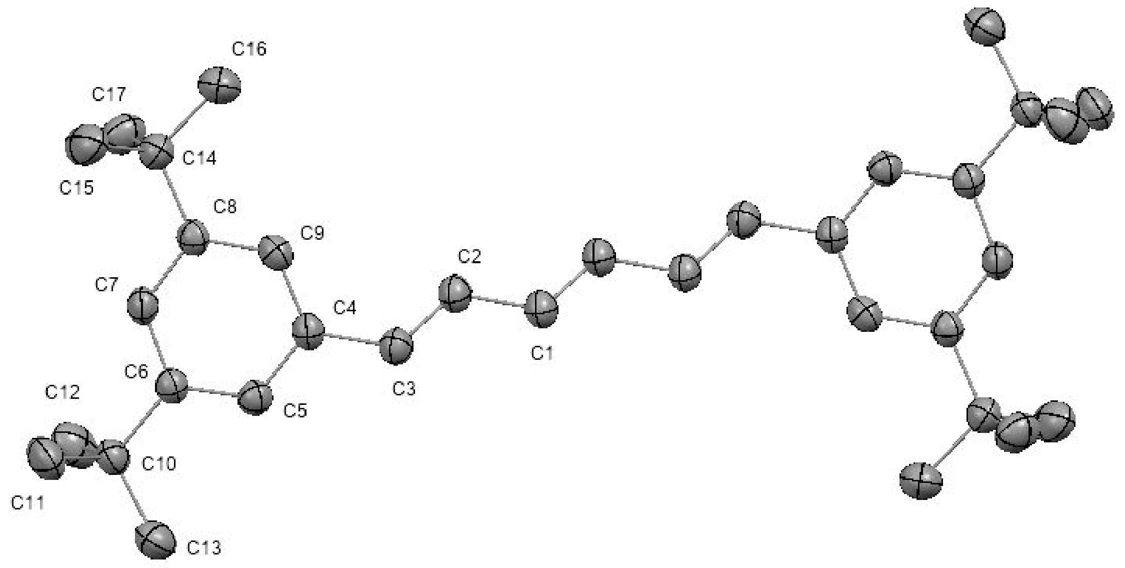

Figure S4: *Molecular structure of* ***DPH1-TTB*** *with displacement ellipsoids at 50% probability. H atoms are omitted. The molecule is situated on an inversion centre in space group* $P2_1/c$*.*

S4- Synthesis and Exchange of Lead-Sulphide QDs

S4.1. Synthesis of PbS-OA QDs

The synthesis of PbS QDs was carried out following modified versions of the method of Hines & Scholes.[31] A typical synthesis was as follows: Lead oxide (0.625 g, 99.999%, Sigma Aldrich), oleic acid (OA, 2 mL, 90%, Sigma Aldrich) and 1- octadecene (ODE, 25 mL, 90%, Sigma Aldrich) were placed in a three-necked round bottomed flask and degassed under vacuum at 110 °C for 2 hours with stirring, forming a colourless solution. Subsequently, the flask was put under nitrogen flow and heated to 115 °C. In a nitrogen glovebox, a syringe was prepared containing ODE (13.9 mL), diphenylphosphine (DPP, 144 µL, 99%, Sigma Aldrich) and bis(trimethylsilyl)sulphide (TMS2S, 296 µL, 95%, Sigma Aldrich). The syringe containing the sulphur precursor was rapidly injected into the reaction flask, which was allowed to cool to 60 °C. The synthesised nanocrystals were twice purified by precipitation with a mix of anhydrous ethanol/1-butanol, centrifugation and resuspension in hexane. The purified QDs were redispersed in toluene for storage in an argon glovebox.

S4.2. Exchange of PbS-OA to PbS-DPH-CA and PbS-C_6

The ligand exchange process was performed in a nitrogen filled glovebox. The PbS-OA QDs were first diluted to a concentration of 20 mg mL^{-1}. The hexanoic acid (C_6) or DTB-DPH-CA (DPH-CA) ligand solution (100 mg mL^{-1} dissolved in tetrahydrofuran, THF) was then added to the PbS-OA QD dispersion, with a QD:ligand mass ratio of 1:0.1. The mixture was stirred for 30 min. The exchanged QDs were purified in three cycles by adding acetone, centrifuging, discarding the supernatant, and redispersing the pellet in toluene. Finally, the QDs were re-dispersed in toluene for film deposition.

S5: Experimental Methods

S5.1. Film preparation

Stock solutions of DPH1-(TTB), DPH2-(TM), DPH3-(TIPO) and DPH4-(BATIPS) were prepared in toluene (60 mg mL^{-1}). Organic host solutions were heated to 50 ºC for 5 min and vortex mixed prior to use. QDs (PbS-OA, PbS-C_6 and PbS-DPH-CA) were re-dispersed in toluene (60 mg mL^{-1}). DPH:QD blends were mixed by volume to prepare solutions containing a total OSC content of 50 mg ml^{-1} and QD contents of 10 mg ml^{-1}. Silicon substrates were cleaned with Decon and ethanol followed by three deionized water rinses. 50 µL of casting solution was deposited on silicon substrates and spin-coated at 1500 rpm for 2 mins. All samples were prepared in a nitrogen glovebox and stored under nitrogen for 24 hrs prior to X-ray scattering measurements.

S5.2. X-ray Scattering

Solution SAXS and GIXS measurements were performed on a Xeuss SAXS/WAXS laboratory beamline (Xenocs) equipped with an liquid gallium MetalJet X-ray source (Excillum), wavelength of characteristic radiation λ = 1.34 Å. Scattering patterns were recorded on a vertically-offset Pilatus 1M detector (Dectris) with a sample to detector distance of 352 mm, calibrated using a silver behenate standard to achieve a q-range of 0.045 – 1.7 Å^{-1}. Two-dimensional images were recorded with exposure times of 600 s.

Solution SAXS samples were measured in 2 mm external diameter borosilicate glass capillaries with a 0.01 mm wall thickness. Data reduction was performed using the instrument-specific Foxtrot software before fitting was performed using SasView in the q range q < 0.4 Å^{-1} using root square scattering intensity data, |*sqrt(I)*| weighting and the Nelder-Mead Simplex algorithm.[8]

For GIXS measurements, films were prepared on silicon substrates and measurements were performed at an incidence angle of 0.3º after. Detector images were corrected, reshaped and reduced using python code which relies on pyFAI and pygix libraries.[7] Azimuthally integrated q-dependent 1D intensity profiles were performed across the full azimuthal angle (χ) and q ranges. Reduced 1D intensity profiles were fitted in SasView software in the q range 0.08 Å^{-1} < q < 0.27 Å^{-1} using root square scattering intensity data, |*sqrt(I)*| weighting and the Nelder-Mead Simplex algorithm.[8]

S6: Solution SAXS

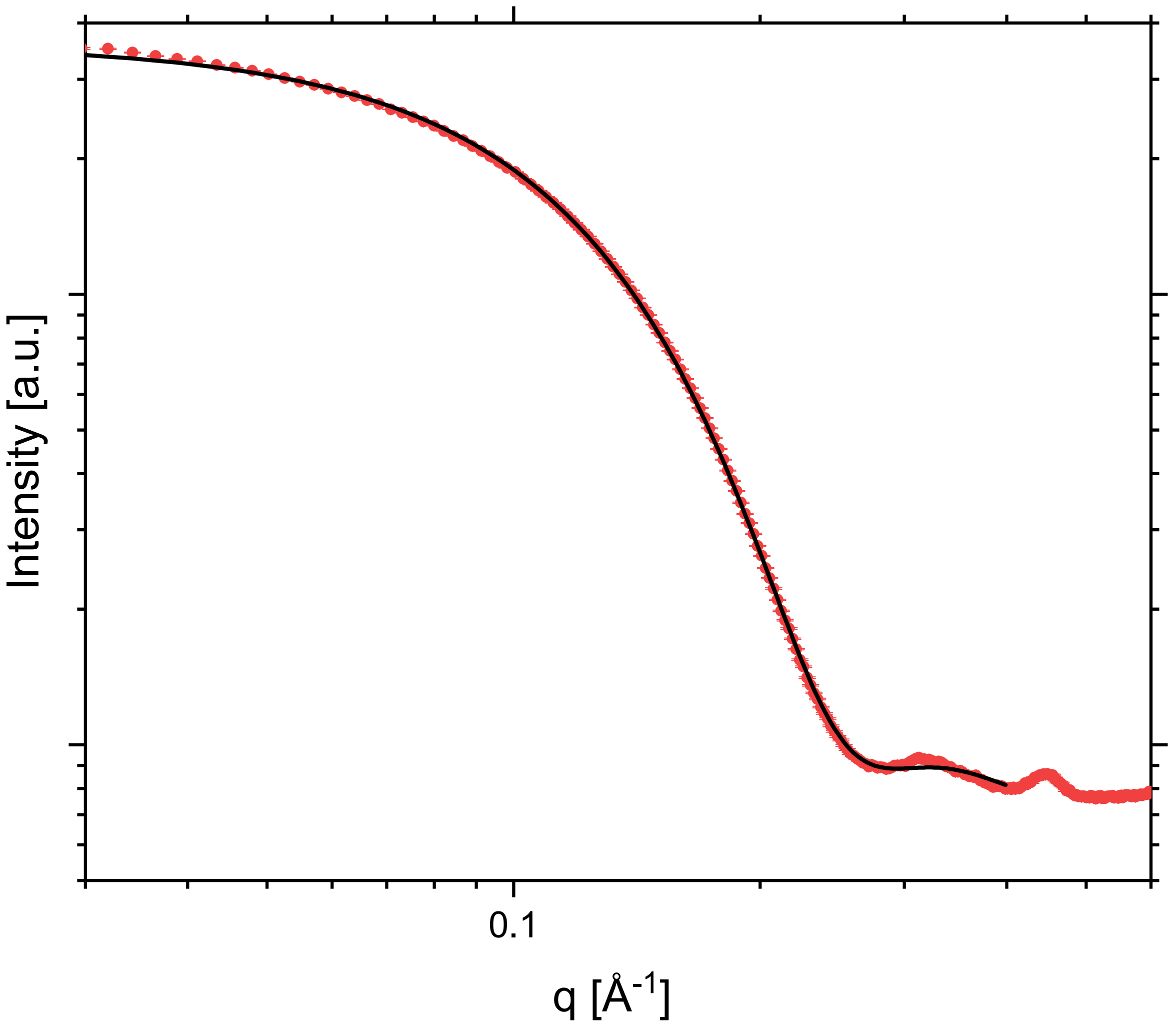

Figure S5: *Azimuthally integrated solution SAXS of as-synthesised PbS-OA QDs in toluene, fitted with a sphere model (solid black lines) in the q-range < 0.4 Å^{-1}.*

Table S2: *Solution SAXS fit parameters from sphere model for as-synthesised PbS-OA QDs in toluene.*

Scale	Background	SLD Sphere [10^{-6}Å^{-2}]	SLD Solvent [10^{-6}Å^{-2}]	Sphere Radius [Å]	Polydispersity
1.033 ± 0.006	72.2 ± 1.2	50.3	10.0	15.673 ± 0.114	0.140 ± 0.005

S7: Single Crystal and Thin Film Crystal Structure Comparison

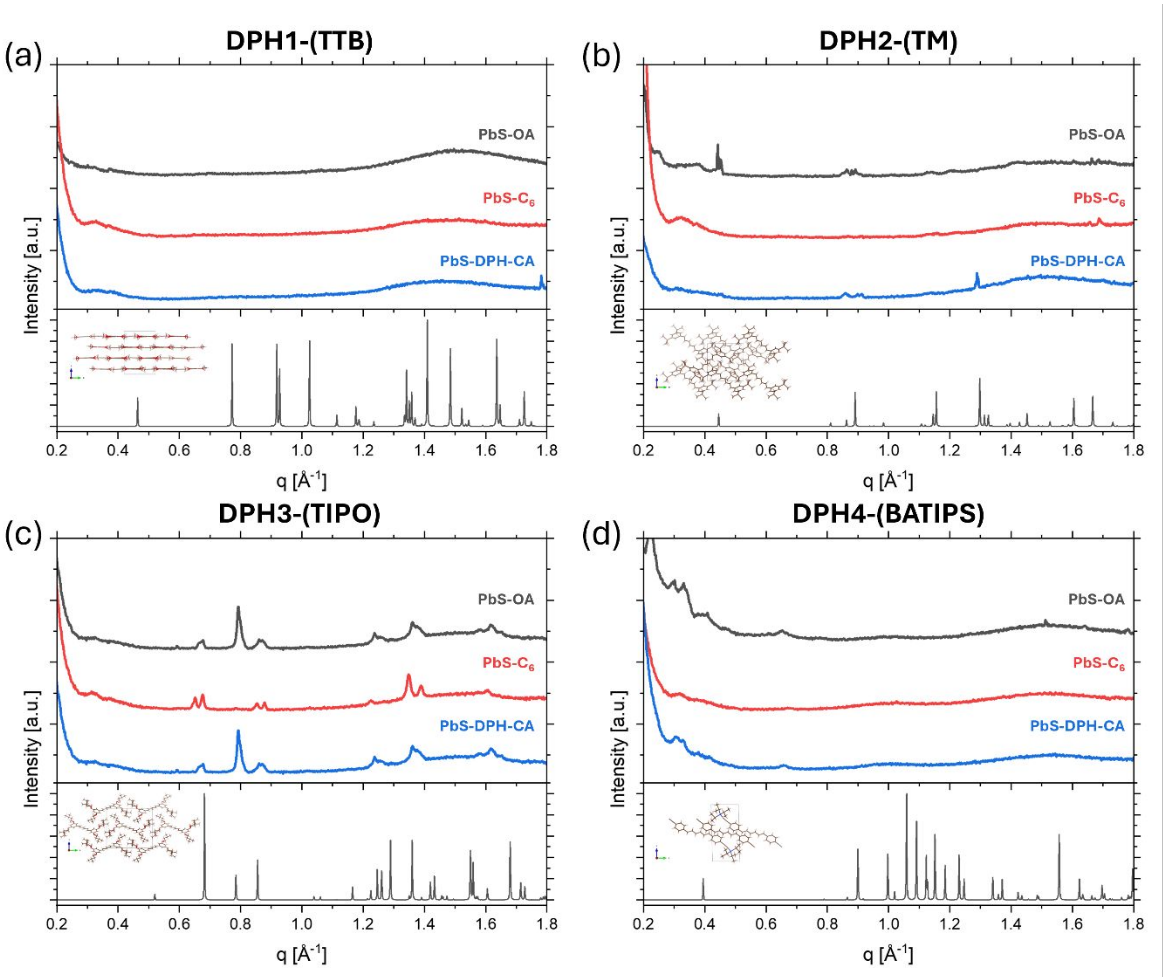

Figure S6: *1D GIWAXS intensity profiles for blends comprising PbS-OA, PbS-C_6 and PbS-DPH-CA QDs with (a) DPH1-(TTB) (fresh), (b) DPH2-(TM), (c) DPH3-(TIPO) and (d) DPH4-(BATIPS) small molecules. Below are powder X-ray diffraction profiles of the small molecule simulated from the single crystal structures as illustrated (Section S3). The 1D data have been multiplied by an arbitrary coefficient to be shifted along the intensity axis for clarity.*

S8: 2D GIWAXS Simulations

2D GIWAXS simulations were generated from the single crystal data (Section S3) using the WAXS package in the SimDiffraction Matlab toolbox.[13] For DPH3-(TIPO):PbS-DPH-CA blends, experimental data was adequately replicated by simulating a crystal arrangement with 002 lattice planes aligned preferentially parallel to the substrate as shown in **Figure S8**. In this orientation, the *c* crystallographic axis is aligned parallel to the out-of-plane direction (q_z direction in reciprocal space). Simulations were generated with a uniaxial model with a Pseudo-Voigt (PSV) distribution using the following SimDiffraction parameters:

- **Reciprocal axis oriented along q_z:** this describes the primary orientation with respect to the substrate [002 for DPH3-(TIPO)].
- **χ angles to probe:** the range of probed crystallite tilts away from the primary orientation (-15° : 1° : 15°).
- **Width of out-of-plane distribution:** describes the width of the PSV distribution ($W_\perp$ = 15°).
- **Line shape of out-of-plane distribution:** the proportion of Gaussian or Lorentzian contribution to the PSV model ($\mu_\perp$ = 0.5).

See the SimDiffraction software manual for further details.[13]

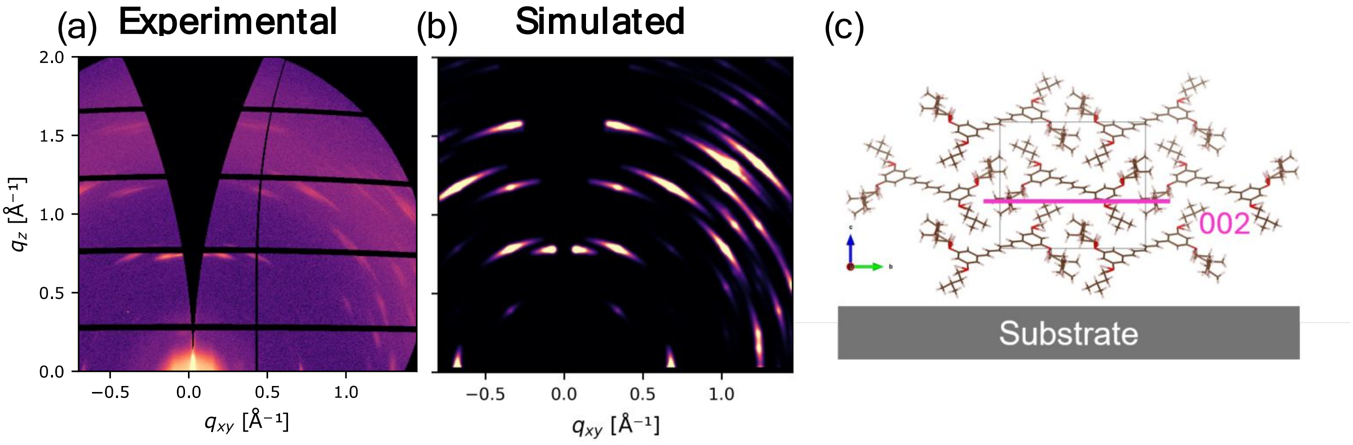

Figure S7: *(a) Experimental 2D GIWAXS pattern of a DPH3-(TIPO):PbS-DPH-CA blend and (b) simulated 2D GIWAXS pattern of DPH3-(TIPO) generated from the single crystal structure with 002 planes aligned parallel to substrate as illustrated (c).*

S9: Colloidal Paracrystal Models

To fit the 1D GISAXS data, we employ a either a face-centered cubic (FCC) or body-centred cubic (BCC) lattice with paracrystalline distortion as outlined in previous works.[9,10]

The FCC and BCC paracrystal models (as implemented in SasView version 6.0.0) calculate the scattering intensity $I(q)$ as:

$$I(q) = \frac{scale}{V_p} V_{lattice} F(q) Z(q) + background$$

where $scale$ is the volume fraction of spheres in the sample, V_p is the volume of the primary particle, $V_{lattice}$ is a volume correction for the crystal structure, $F(q)$ is the form factor of the sphere (normalized), and $Z(q)$ is the paracrystalline structure factor for an FCC or BCC structure.[8,11,12] GIXS data in this work is not calibrated to an absolute intensity scale, as such, the 'scale' and 'background' terms represent a convolution of instrumental factors, sample thickness, sample transmission and baseline offsets. They are included to improve fit quality but have no standalone physical meaning.

S9.1 - FCC Paracrystal Model

The lattice correction (the occupied volume of the lattice) for an FCC structure of particles of

radius R and the nearest neighbour separation D is:

$$V_{lattice} = \frac{8\pi R^3}{3\sqrt{2}D^3}$$

The distortion factor (one standard deviation) of the paracrystal is included in the calculation of $Z(q)$

$$\Delta a = gD$$

where g is a fractional distortion based on the nearest neighbor distance.[11]

S9.2 - BCC Paracrystal Model

The lattice correction (the occupied volume of the lattice) for an BCC structure of particles of

radius R and the nearest neighbour separation D is

$$V_{lattice} = \frac{\sqrt{3}\pi R^3}{D^3}$$

The distortion factor (one standard deviation) of the paracrystal is included in the calculation of $Z(q)$

$$\Delta a = gD$$

where g is a fractional distortion based on the nearest neighbor distance.[12]

S9.3 – Simulated FCC and BCC 1D Profiles

To better visualise the effect of the disorder parameter on QD ordering, simulated FCC and BCC paracrystal models for QDs with a radius of 16 Å are presented in Supplementary Figure S8. Here, highly ordered QDs are characterised by low disorder parameters corresponding to a highly aggregated FCC or BCC arrangement. In contrast, weakly ordered QD arrangements are characterised by low disorder parameters where a value of 1.0 corresponds to a random QD arrangement described by a scattering model of spheres with hard-sphere interactions.

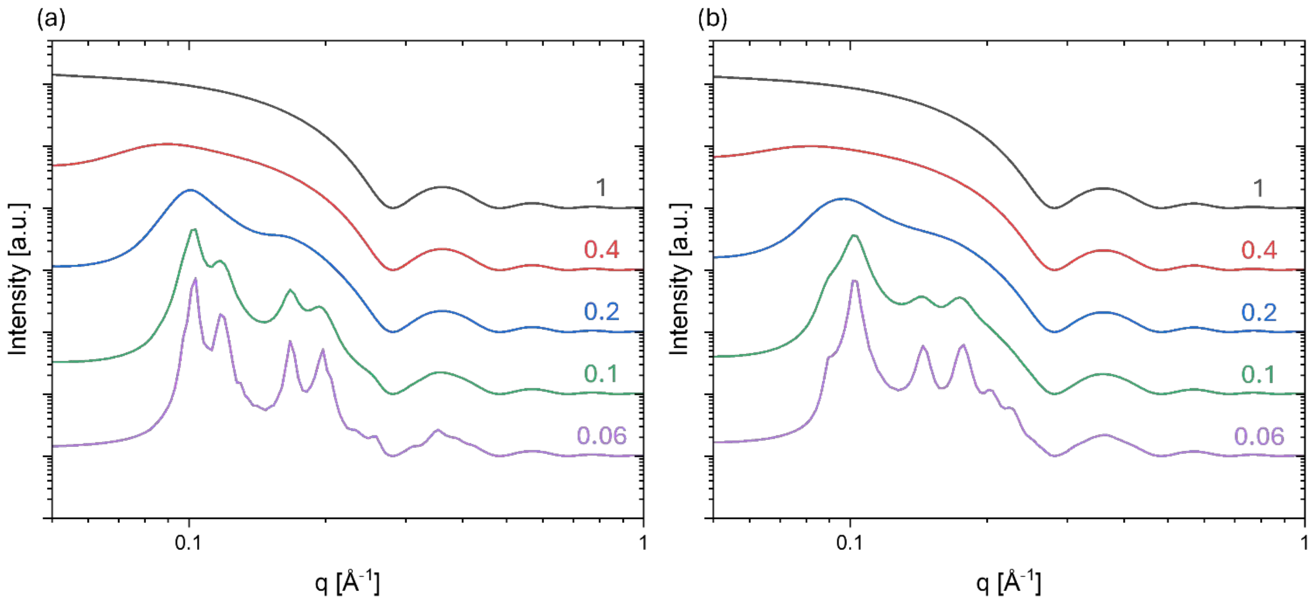

Figure S8: *The effect of disorder parameter on the packing of QDs using the (a) FCC and (b) BCC paracrystal model. The 1D data have been multiplied by an arbitrary coefficient to be shifted along the intensity axis for clarity.*

S10: 1D GISAXS Fitting

Table S3: *Fit parameters of QD structures for QD:small molecule blend films with PbS QDs (possessing either; oleic acid, hexanoic acid or a DPH-carboxylic acid derivative ligands) and various DPH small molecule host species [DPH-(TTB/TM/TIPO/BATIPS)]. Data fitted using an FCC or BCC paracrystal model. The following parameters were fixed: Radius=16 Å, QD particle SLD=50.30 x10^{-6} Å^{-2} and solvent SLD = 10 x10^{-6} Å^{-2}.*

QD	Organic small molecule	Paracrystal Model	Scale	Background	Lattice constant [Å]	Disorder parameter
PbS-OA	DPH1-(TTB)	FCC	0.19	5.65	45.2	0.61
	DPH2-(TM)	BCC	1.31	6.17	52.8	0.09
	DPH3-(TIPO)	FCC	1.39	7.02	55.1	0.16
	DPH4-(BATIPS)	FCC	1.25	39.1	55.4	0.14
PbS-C_6	DPH1-(TTB)	FCC	3.27	5.83	76.4	1.00
	DPH2-(TM)	BCC	0.90	9.18	40.5	0.16
	DPH3-(TIPO)	FCC	0.44	2.49	35.8	0.44
	DPH4-(BATIPS)	FCC	0.31	13.7	32.5	0.53
PbS-DPH-CA	DPH1-(TTB)	FCC	3.15	4.71	80.4	1.00
	DPH2-(TM)	BCC	0.61	8.96	50.0	0.14
	DPH3-(TIPO)	FCC	0.54	2.99	44.4	0.57
	DPH4-(BATIPS)	FCC	0.58	3.70	43.4	0.60

S11: Aged DPH1-(TTB):QD Films

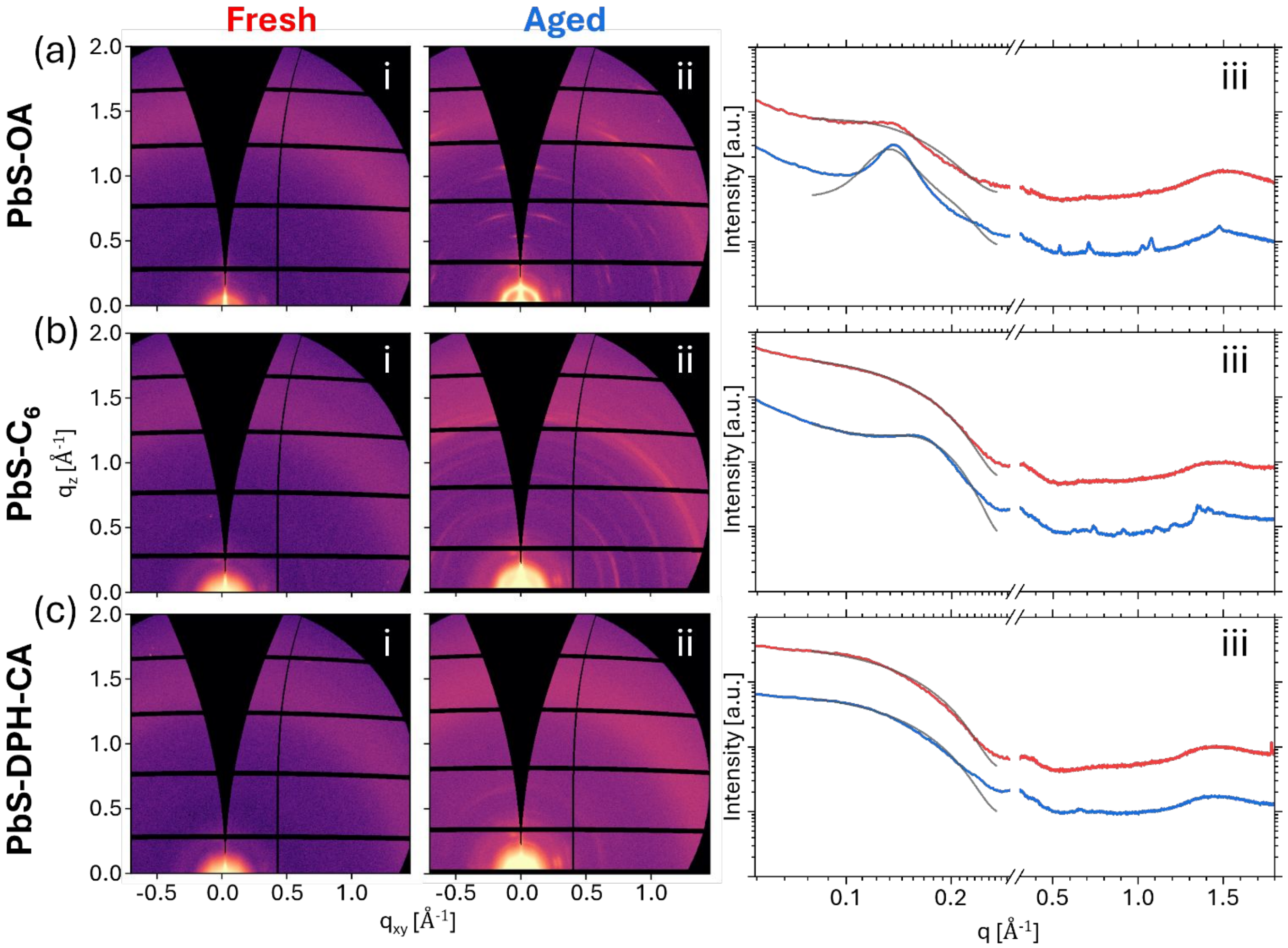

Figure S9: *2D GISAXS patterns of OSC:QD blends comprising DPH1-(TTB) and (a) PbS-OA, (b) PbS-C_6 or (c) PbS-DPH-CA, measured (i) fresh and (ii) after 2 weeks of ageing under dark, ambient conditions. (iii) Corresponding 1D azimuthally integrated data showing fresh (red line), aged (blue line), and the associated fits (grey lines) using an FCC paracrystal model. The 1D data have been multiplied by an arbitrary coefficient to be shifted along the intensity axis for clarity.*

Table S4: *Fit parameters of QD structures for fresh and aged blend films comprising DPH1-(TTB) and with PbS possessing either; oleic acid, hexanoic acid or a DPH-carboxylic acid derivative ligands. Data fitted using an FCC paracrystal model. The following parameters were fixed: Radius = 16 Å, QD particle SLD = 50.30 x10*$^{-6}$ *Å*$^{-2}$ *and solvent SLD = 10 x10*$^{-6}$ *Å*$^{-2}$*.*

QD	Film Condition	Scale	Background	Lattice constant [Å]	Disorder parameter
PbS-OA	Fresh	0.19	5.65	45.2	0.61
	Aged	1.07	8.64	54.78	0.25
PbS-C_6	Fresh	3.27	5.83	76.4	1.00
	Aged	0.83	7.60	35.7	0.43
PbS-DPH-CA	Fresh	3.15	4.71	80.4	1.00
	Aged	9.14	9.51	93.2	0.51

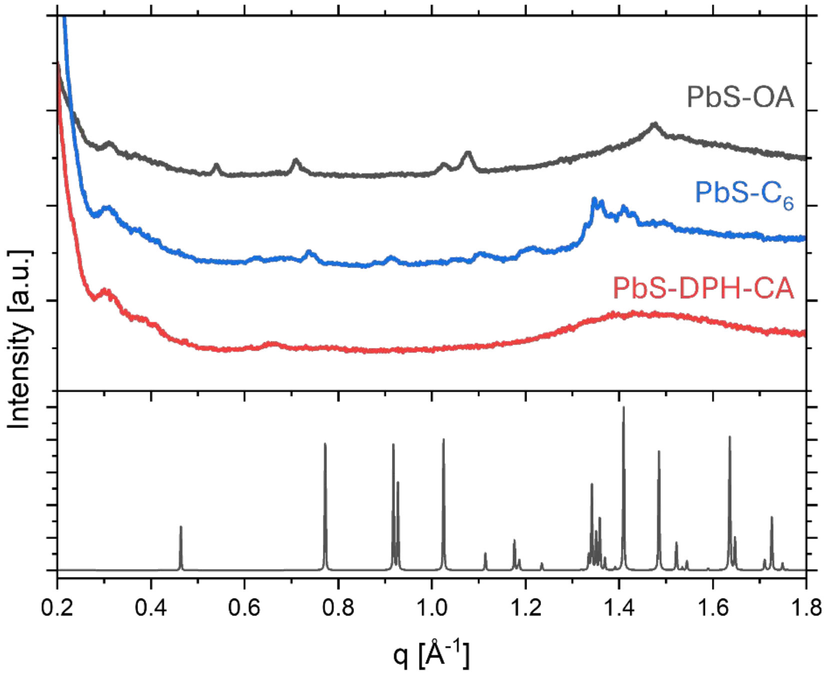

Figure S10: *1D GIWAXS intensity profiles of aged blends comprising PbS-OA, PbS-*C_6 *or PbS-DPH-CA QDs with DPH1-(TTB). Below is a powder X-ray diffraction profile of DPH1-(TTB) simulated from the single crystal structure (Section S3). The data are offset for clarity.*

S12: References

1. Pfiffi, D., Bier, B. A., Marian, C. M., Schaper, K. & Seidel, C. A. M. Diphenylhexatrienes as Photoprotective Agents for Ultrasensitive Fluorescence Detection. *J. Phys. Chem. A* **114**, 4099–4108 (2010).

2. Drew, J., Letellier, M., Morand, P. & Szabo, A. G. Synthesis from pregnenolone of fluorescent cholesterol analog probes with conjugated unsaturation in the side chain. *J. Org. Chem.* **52**, 4047–4052 (1987).

3. Brett Runge, M., Mwangi, M. T. & Bowden, N. B. New selectivities from old catalysts. Occlusion of Grubbs' catalysts in PDMS to change their reactions. *J. Organomet. Chem.* **691**, 5278–5288 (2006).

4. Nierengarten, J.-F. *et al.* Synthesis and Optical Properties of Isomeric Branched π-Conjugated Systems. *J. Org. Chem.* **70**, 7550–7557 (2005).

5. Challa, C., Vellekkatt, J., Ravindran, J. & Lankalapalli, R. S. A metal-free one-pot cascade synthesis of highly functionalized biaryl-2-carbaldehydes. *Org. Biomol. Chem.* **12**, 8588–8592 (2014).

6. Khurana, J. M., Chauhan, S. & Bansal, G. Facile Hydrolysis of Esters with KOH-Methanol at Ambient Temperature. *Monatshefte für Chemie / Chem. Mon.* **135**, 83–87 (2004).

7. Ashiotis, G. *et al.* The fast azimuthal integration Python library: pyFAI. *J. Appl. Crystallogr.* **48**, 510–519 (2015).

8. SasView - Small Angle Scattering Analysis. https://www.sasview.org/. [Date accessed: 05/12/2025]

9. Toolan, D. T. W. *et al.* Insights into the kinetics and self-assembly order of small-molecule organic semiconductor/quantum dot blends during blade coating. *Nanoscale Horiz.* **8**, 1090–1097 (2023).

10. Gray, V. *et al.* Ligand-Directed Self-Assembly of Organic-Semiconductor/Quantum-Dot Blend Films Enables Efficient Triplet Exciton-Photon Conversion. *J. Am. Chem. Soc.* **146**, 7763–7770 (2024).

11. SasView - FCC Paracrystal. https://www.sasview.org/docs/user/models/fcc_paracrystal.html. [Date accessed: 05/12/2025]

12. SasView - BCC Paracrystal. https://www.sasview.org/docs/user/models/bcc_paracrystal.html. [Date accessed: 05/12/2025]

13. Breiby, D. W., Bunk, O., Andreasen, J. W., Lemke, H. T. & Nielsen, M. M. Simulating X-ray diffraction of textured films. *J. Appl. Crystallogr.* **41**, 262–271 (2008).
